# Psychopathic personality traits stress immunity and social potency moderate the relationship between emotional competence and cognitive functions in depression

**DOI:** 10.3389/fpsyt.2023.1061642

**Published:** 2023-03-27

**Authors:** Elena M. D. Schönthaler, Nina Dalkner, Karin Schwalsberger, Eva Z. Reininghaus, Bernd Reininghaus

**Affiliations:** Department of Psychiatry and Psychotherapeutic Medicine, Medical University Graz, Graz, Austria

**Keywords:** psychopathic personality traits, stress immunity, social potency, depression, cognitive functions, emotional competence, affective disorders, personality

## Abstract

**Background:**

Psychopathic personality traits (PPT) and depression have both been shown to worsen emotional and cognitive functions. Moreover, PPT and depression share similar underlying neuronal circuits tapping into the emotional and cognitive domains. However, little is known about the influence of PPT on emotion and cognition in individuals with depression.

**Objectives:**

This study aimed to examine the correlative relationships and moderating role of PPT in the association between emotional competence and cognitive functions in individuals with depression.

**Methods:**

Data from 373 individuals diagnosed with depression (158 males, 215 females) were examined within a cohort study. Subjects filled out validated questionnaires surveying PPT and emotional competences. Furthermore, a comprehensive neuropsychological test battery was administered.

**Results:**

Correlation analyses revealed a significant positive association between emotional competence and cognitive functions. Further, negative associations between emotional competence and the PPT “Blame Externalisation” and “Careless Nonplanfulness,” as well as positive associations with psychopathic “Social Potency” and “Stress Immunity” were found. Moderation analyses indicated a significant positive influence of psychopathic “Stress Immunity” and “Social Influence” on the relationship between emotional competence and cognitive functions.

**Conclusion:**

The findings highlight the importance of integrating PPT in depression research. Considering PPT in depression treatment could also facilitate the therapeutic process by identifying individual traits as resilience-strengthening or potentially harmful factors for depressive symptomatology. This study represents a stepping stone for further research regarding the role of personality traits in psychiatric disorders and their treatment.

## 1. Introduction

Depression is an affective disorder encompassing depressed mood, diminished interests and joy, deteriorated cognitive function, and accompanying vegetative symptoms, such as sleep or appetite disturbances (1). Depressive symptoms are thus mainly found in emotional and cognitive domains. For example, recent research indicated that individuals with depression show reduced emotional reactivity to aversive stimuli (2), difficulties in emotion regulation (e.g., 3, 4), or a negative response bias toward facial emotion expressions (5). At the same time, better abilities to regulate emotions have been connected to greater resilience to depression following traumatic experiences (6). Regarding cognitive functioning, there is accumulating evidence suggesting cognitive deterioration in individuals with depression; for example, depression has been associated with cognitive deficits in executive function, attention (7), psychomotor speed, learning, visual memory (8), working memory, and long-term memory (9). The connection between cognitive functioning and emotion in depression has also been examined. A review across several studies on depression, cognition, and emotion regulation indicated cognitive biases (in attention, memory, and cognitive control) toward negative stimuli in depression, which lead to an impaired ability in reinterpreting situations more adaptively (e.g., reappraisal) and instead an increased use of maladaptive cognitive strategies (e.g., rumination; 3). Depressive symptoms can be assigned to many different aspects, factors contributing to their development are, for example, environmental aspects (e.g., childhood abuse and stressful events) and heritability (1). Nevertheless, no single mechanism can fully explain all aspects of depression, thus several factors need to be considered in its etiology. Personality traits have been shown to contribute to the development of depression (e.g., 10).

Psychopathic personality traits (PPT) are characterized by affective, behavioral, and interpersonal features, such as egocentricity, non-sympathetic behavior, and a tendency to violate social and legal norms (11). They circumcise many of the same traits found in antisocial personality disorder (APD), which is recognized by early antisocial actions, recklessly disregarding other’s rights, and a continuous demonstration of impulsive and irresponsible behavior. In addition to the characteristics seen in APD, PPT comprise affective and interpersonal qualities, such as superficial charm, deceptive, and manipulative behavior toward others, callousness, and lack of guilt, empathy, or remorse. Thus, most individuals with pronounced PPT fulfill the criteria for APD, but not all individuals diagnosed with APD meet the criteria for PPT (12). Notably, PPT criteria are found in APD, but can also be retributed to narcissistic, histrionic, and borderline personality disorders, thus including various traits of different personality disorders (13, 14). Similar to depression, PPT is also characterized by emotional and cognitive functioning deficits. For example, studies indicated that individuals with higher PPT scores tend to lack the ability to regulate or process their own emotions (15–18), focus on emotional stimuli (19), or differentiate correctly between emotional aspects of facial expressions (20, 21). Similarly, evidence from meta-analytic work reveals deficits in recognizing emotions in faces or vocals of others in individuals scoring high in PPT measures (22, 23). However, there are also findings indicating no difference in emotion perception and responsiveness between individuals with higher or lower PPT scores (24). Regarding cognitive functions, there is profound evidence on the negative relationship between PPT and certain aspects of attention (25–28), executive function (29, 30), and other cognitive processes such as language processing, which is, among others, attracted to underlying impaired neurobiological processes (31).

The connection between depression and PPT was examined in previous studies, with results indicating mainly positive associations between PPT and depression or cognitive and interpersonal aspects of depression (e.g., 32, 33). However, other studies revealed inverse relationships between PPT and depression, pointing toward a protective function of PPT in depression (e.g., 34–36). The role of PPT in depression has thus yet not been clearly determined; however, both constructs share the feature of deteriorating influences on emotion and cognitive functioning. This conclusion is also supported by evidence from neurobiological research, which indicates altered activity and connectivity in the regions commonly found to be impaired in depression and anxiety (e.g., the frontal lobe circuitry, 37–40). For example, functional changes in brain circuits targeting the cognitive control network and the affective-salience network were found in individuals high in PPT. In individuals with higher PPT, disturbances in the neurobiological stress-response systems, including the hypothalamic–pituitary–adrenal axis, and the immune system were identified (1). Studies on structural brain alterations in depression found altered regional brain volumes such as a hippocampal atrophy (41, 42), enlarged amygdala volume (43, 44), alterations in the prefrontal cortex (45) and anterior cingulate cortex (46), all of which play a major role in emotion and cognitive functioning (e.g., 47). In line with the alterations found in individuals with depression, research on neurobiological correlates of PPT showed impairments in functions tapping into emotional and cognitive domains (48, 49). For example, studies found a reduction of gray matter volume in prefrontal and temporal areas, including the orbital frontal cortex, anterior cingulate cortex, as well as amygdala and hippocampus alterations (31, 50, 51). Functionally, a systematic review indicated that PPT are related to a dysfunction of the default mode network, which is involved with moral judgment, metacognitive and introspective abilities (52). Moreover, PPT are assumed to be associated with imbalances in the neurotransmitter system (53), among others they are connected to serotonin pathways (54, 55), which also have been shown to be altered in depression (e.g., 56). In PPT, it is supposed that low serotonin levels interact with testosterone, resulting in increased violent and impulsive behavior. The ratio between testosterone and cortisol is also assumed to be imbalanced in pronounced PPT, thus resulting in emotional deficits (57, 58). Interestingly, alterations in cortisol levels were also found in depression and connected to cognitive deficits and emotional processing within the disease (59, 60). Finally, altered autonomic responses to emotional stimuli have been found in both PPT (61) and depression (62).

Current research depicts the connection between depression and PPT, as well as their deteriorating influence on emotion and cognitive functioning. Both depression and PPT seem to share underlying neurobiological processes, which are connected to emotion and cognition processes. The connection between emotional, cognitive, and personality domains is also supported by transdiagnostic models like the Research Domain Criteria (RDoC) constructs, which describe a neurobiological foundation of human experience. In the RDoC framework, psychological constructs are categorized in five main domains: Negative Valence Systems, Positive Valence Systems, Cognitive Systems, Social Processes, and Arousal and Regulatory Systems. Each domain is further divided into constructs and subconstructs, which may be further examined using seven units of analysis. It was found that personality aberrations are tied to RDoC constructs in the Social Processes domain, indicating that there are brain circuits involved in facial emotion perception and interpersonal rejection. Further, the RDoC domains of Negative and Positive Valence were found to be associated with clinical personality disorders, thus reflecting the disruption of computational processes involved in estimating risks and benefits of a future outcome. Next to the social and emotional RDoC domains, the Cognitive Systems domain, especially the subconstruct “Cognitive Control” has been shown to be related to personality disorders associated with impulse control, thereby suggesting a connection of clinical personality disorders to neural circuits influencing goal selection and behavioral control. Finally, personality disorders were also connected to the RDoC domain of Arousal and Regulatory Systems, indicating a biological sensitivity to internal and external stimuli (63). In psychopathological illnesses like depression, these transdiagnostic approaches between emotion, cognition, and personality are specifically important. Bifactor models, in which a general factor of psychopathology is specified overarching externalizing and internalizing factors, were previously associated with the Big Five personality traits (64), and maladaptive personality traits on multiple levels of the hierarchical taxonomy of psychopathology (HiTOP) structure, which also represents a hierarchical transdiagnostic model (65). Derived from the HiTOP dimensional model of psychopathology, it has been suggested that personality traits have substantial implications for later psychopathology (66), and thus associated functions like emotion and cognition.

This leads to the question of what role PPT play in the development and maintenance of depressive symptomatology, specifically in the emotional and cognitive domains. The current study thus aimed to examine the influence of PPT on the relationship between emotional competence and cognitive function in individuals with depression. Based on current literature, we hypothesized that (a) there is a significant positive relationship between emotional competence and cognitive functions in individuals with depression, (b) PPT are significantly related to emotional competence and cognitive functions, and (c) PPT significantly moderate the relationship between emotional competence and cognitive functions in individuals with depression.

## 2. Materials and methods

### 2.1. Sample

Data from 1,447 individuals with psychiatric disorders were collected between April 2015 and February 2017 at an Austrian psychiatric rehabilitation center focusing on affective and stress-related diseases. For this study, we only included individuals fulfilling the diagnostic criteria for depression (independently of depression severity or presence of current episode) above the age of 18 years. Six hundred and twenty-three participants who were already diagnosed according to the ICD-10 criteria before their rehabilitation stay by experts working in the psychiatric and psychological field were included in the current analysis. However, due to missing data in the questionnaires examining PPT, emotional competence, and cognitive functions, data of 250 participants were excluded. Further exclusion criteria were a comorbid disorder of schizophrenia, neurodegenerative diseases, acute psychotic symptoms, or substance abuse, since the latter is followed by altered cognitive functions in the domains of attention, inhibition, working memory, and decision-making (67). However, none of the remaining subjects fulfilled these criteria. Participants were also screened for neurological diseases, but were not excluded in case they had neurological illnesses. In total, 373 individuals (158 male, 215 female) with a mean age of 52.64 (*SD* = 7.50) were included in all analyses. A *post hoc* power analysis (using GPower, version 3.4; 68) indicated a sufficiently large sample size (*n* ≥ 325) to conduct moderated regression analyses and detect a small effect (*f*^2^ = 0.03), considering an α-level of 0.05 and a power of 80%. Participants provided written informed consent prior to taking part in the study. This study was conducted in accordance with the Declaration of Helsinki and was provided with a positive ethics vote of the ethics committee of the Federal State Upper Austria (EK-number: E-24-14).

### 2.2. Material

This study was part of a large-scaled study focusing on the investigation of several psychological, biological, and cognitive parameters in psychiatric rehabilitation treatment (for detailed description see also 34, 69–71). Only measures which were assessed in the beginning of the treatment (including PPT, emotional, and cognitive parameters) were relevant for the present investigation as described in the following. In total, the study completion took at least two hours.

#### 2.2.1. Emotional competence

Emotional competence was assessed with the German version of the Emotional Competence Questionnaire (72). This self-report questionnaire includes 62 items allocated to four scales (Recognizing and Understanding Emotions, Recognizing Emotions of Others, Regulation and Control of own Emotions, and Emotional Expressivity). Items are presented as statements (e.g., “When I’m on the phone with a friend, I can understand what he’s feeling”). Subjects were asked to answer the items on a five-point Likert scale, ranging from (1) “strongly disagree” to (5) “strongly agree.” Scale scores were built by calculating the mean of the corresponding items. Moreover, a total score was created by calculating the mean of all scales (ECQ Score).

#### 2.2.2. Psychopathic personality traits

Psychopathic personality traits were measured with the German version of the Psychopathic Personality Inventory-Revised (PPI-R; 73; Original version: 74). This self-report questionnaire contains 154 items offering scores for eight subscales: (1) “Machiavellian Egocentricity” (measures ruthlessness and an intention to manipulate others), (2) “Social Potency” (measures superficial charm and striving for interpersonal dominance), (3) “Coldheartedness” (records callousness and lack of guilt), (4) “Carefree Nonplanfulness” (measures deficiencies in planning behavior and controlling maladaptive impulses), (5) “Fearlessness” (measures a tendency for high-risk behavior), (6) “Blame Externalization” (records externalizing own blame), (7) “Impulsive Nonconformity” (measures indifference toward social customs), and (8) “Stress Immunity” (records the lack of emotional reactions toward possibly fear-inducing situations; 75). Items are presented as statements (e.g., “I use many white lies”). Subjects were asked to answer on a four-point Likert scale [(1) = “False,” (2) = “Mostly False,” (3) = “Mostly True,” and (4) = “True”]. Subscores and a total PPT score were built by creating the mean of the corresponding items.

#### 2.2.3. Cognitive domains

##### 2.2.3.1. Attention and processing speed

To calculate the cognitive domain score of attention and processing speed, we used the scores of the Trail Making Test part A (TMT A; 76), in which participants were asked to connect digits in an ascending order. Further, we used the word- and color-naming trials from the Stroop Color and Word Interference Test (77), in which participants were asked to read aloud a list of color words printed in black, a list of color words printed in the color of the word itself, and a list of color words printed in a color diverging from the color word (interference trial). Moreover, the revised d2-Test (d2-R; 78) was administered to measure attention, focus efficiency, and accuracy during the distinction of visually similar objects. Participants were asked to mark the letter “d” with two dashes but no other distractors in several rows of letters.

##### 2.2.3.2. Verbal learning and memory

To construct the domain of verbal learning and memory verbal learning and memory, we administered the German version of the California Verbal Learning Test (CVLT; 79). In this test, participants were instructed to memorize a list of 16 nouns drawn from four semantic categories, which was repeated in fixed order five times (list A). They were asked to recall as many words as possible in any order after each repetition (recall trials 1–5). Hereafter, a list of distractor words was presented to them and they were asked to freely recall the distractor words (list B). Free and cued recall of list A were then tested immediately (short-delay free recall, short-cued recall), and again after 20 min (long-delay free recall, long-delay cued recall). In the cued recall, the subjects were prompted with the semantic word category. Finally, subjects were presented with a recognition task, which encompassed 44 words either from list A or other distractor lists. For each word, participants had to indicate whether it stems from list A or is a distractor word. We included all free and cued recall trials from list A into the verbal memory domain score. Moreover, the Digit Span Test (forward recall), which is part of the Wechsler Adult Intelligence Scale 4th Edition (WAIS-IV; 80), was administered. In this test, participants were asked to recall number sequences that were read to them in the same order.

##### 2.2.3.3. Executive function

To assess the executive function domain, we recorded scores of the TMT B, in which participants had to connect digits and letters in ascending order (76), and calculated the difference between TMT B and TMT A. This difference indicates task switching ability (78). Further, we considered the interference trial of Stroop Color and Word Interference Test (77), and the Digit Span Test (backward recall), in which participants were asked to recall number sequences that were read to them backward (79).

#### 2.2.4. Beck depression inventory

To descriptively determine depression severity, we administered the Beck Depression Inventory II (BDI-II; 81). Severity of depression is measured with 21 items on a four-point ascending Likert type scoring system. The total sum score ranges from 0 to 63, with higher values indicating higher severity. Clinically significant depression is indicated by scores over 18.

#### 2.2.5. Clinical Global Impression Scale

The Clinical Global Impression Scale was administered to descriptively evaluate clinical severity and course of the current depressive episode. It subsumes three components, i.e., measurement of illness severity, global improvement, and an efficacy index, which records medication efficacy. We used the illness severity component, which is assigned a score between 1 and 7 (1 = “not at all ill,” 7 = “most extremely ill”; 82).

### 2.3. Statistical analyses

Data were analyzed using the softwares SPSS (Version 27, for calculating the cognitive domain scores) and R (www.r-project.org; Version 4.1.2, for all further analyses). To construct scores in the cognitive domains’ attention, executive functioning, and verbal learning/memory, we used the procedure proposed by previous literature (83): First, variables with smaller values indicating better results were multiplied by −1, with the aim of all variables having larger values indicating better performance. Secondly, all variables were transformed to standardized *z*-scores. Third, the newly built *z*-scores were summed up for each of the neuropsychological measures according to their allocation toward the three domains as presented in Table 1. Since these scores were not normally distributed, they were once again *z*-transformed using Rankit’s formula. The mean of all scores was then calculated to build a total cognitive composite score (in the following referred to as composite score).

**Table 1:** Allocation of neuropsychological tests to cognitive domains (83).

Domain	Ability	Neuropsychological test
Attention	Psychomotor speed	TMT A (time in seconds)
		Stroop test word naming (time in seconds)
		Stroop test color naming (time in seconds)
		Digit-symbol-test (number of correct symbols)
Executive function	Working memory	Digit-span backward (correct numbers)
		Stroop test word color interference (time in seconds)
	Task switching	TMT B and TMT B-TMT A (time in seconds)
Verbal learning and memory	Verbal learning	CVLT correct (short-delay free recall)
	Consolidation	CVLT loss of recalled words (short-delay cued recall)
	Long-term memory	CVLT loss of recalled words (long-delay free recall)
	Recognition	CVLT recognition (long-delay cued recall)
	Short-term memory	Digit-Span forward (correct numbers)

TMT A/B, Trail-making-test; CVLT, California verbal learning task.

To test the first and second hypothesis, we conducted bivariate Pearson correlation analyses between the ECQ score, composite score, and PPT subscales using the Benjamini–Yekutieli adjustment for multiple comparisons to control the false discovery rate (84). Moreover, we conducted moderation analyses with 95% BCa-bootstrapping confidence intervals based on 10,000 samples to examine the third hypothesis using the PROCESS macro in R by Hayes (Version v4.0, 85). Assumptions necessary to conduct this analysis were analyzed (linearity, homoscedasticity, multicollinearity, normality, and independence of residuals; see Supplementary material). For all moderation calculations, we centered means of the predictor and moderator variable prior to analyses. To determine whether the gradient of one or both regression slopes per moderation significantly differs from zero, we conducted simple slope analyses. Data and data codes can be accessed *via* https://doi.org/10.17605/OSF.IO/5GUD9.

## 3. Results

### 3.1. Sample description

Table 2 provides information on the sample characteristics.

**Table 2:** Socio-demographic, medical, and psychological sample.

Characteristics	
Sex	
Female	215 (57.64%)
Male	158 (42.36%)
Education	
No formal education	3 (0.80%)
Formal education	44 (11.81%)
Polytechnic education	41 (10.99%)
Middle school diploma	34 (9.12%)
High school diploma	102 (27.35%)
Subject-specific high school diploma	29 (7.77%)
University degree	66 (17.69%)
Other	46 (12.33%)
Not specified	8 (2.14%)
Occupation	
Unemployed	7 (1.88%)
Student	3 (0.80%)
Employed	289 (77.48%)
Self-employed	3 (0.80%)
Retired	10 (2.68%)
Not specified	61 (16.35%)
Psychiatric medication (yes)	314 (84.18%)
Somatic medication (yes)	215 (57.64%)
Neurological disease (yes)	59 (15.82%)
Not specified	52 (13.94%)
Age	*M* = 52.64
	*SD* = 7.50
BDI-II score	*M* = 21.08
	*SD* = 10.35
CGI-score	*M* = 3.61
	*SD* = 1.00
PPI-R	
BE	*M = 32.27*
	*SD* = 8.65
RN	*M* = 48.60
	*SD* = 10.89
SI	*M = 33.67*
	*SD* = 7.15
SP	*M = 38.11*
	*SD* = 8.50
CN	*M* = 30.00
	*SD* = 5.78
ME	*M* = 34.92
	*SD* = 5.55
CH	*M* = 27.93
	*SD* = 5.77
F	*M* = 14.13
	*SD* = 5.08

M, mean; SD, standard deviation; BDI-II score, Beck Depression Inventory II-score. CGI–score, Clinical Global Impression Scale. PPI-R, Psychopathic Personality Inventory-Revised; BE, blame externalization; RN, rebellious nonconformity; SI, stress immunity; SP, social potency; CN, careless nonplanfulness; ME, machiavellian egocentricity; CH, coldheartedness; F, fearlessness.

### 3.2. Correlation analyses

Results on the descriptively conducted bivariate Pearson/Spearman correlations between the demographic characteristics and the study variables can be found in Table 3.

**Table 3:** Bivariate Pearson- and Spearman correlations between demographic characteristics and study variables.

Variable	ECQ	BE	RN	SI	SP	CN	ME	CH	F	CogScore
1. Age	0.04	−0.12	−0.05	0.04	0.03	0.04	−0.04	−0.04	**−0.28** ^*^	**−0.25** ^*^
2. Sex	**−0.20** ^**^	0.08	0.12	0.13	0.05	−0.04	0.11	**0.28** ^*^	**0.35** ^*^	**−0.27** ^*^
3. Education^a^	**0.21** ^**^	−0.13	−0.06	0.08	0.12	−0.07	0.00	−0.09	−0.02	0.14
4. Occupation^a^	0.02	0.01	0.10	0.07	0.14	0.01	0.10	−0.06	−0.01	0.02
5. Psychiatric medication^b^	**−0.17** ^**^	0.07	−0.01	−0.13	−0.14	0.04	0.04	−0.00	−0.04	−0.12
6. Somatic medication^b^	−0.05	0.08	0.00	−0.07	−0.03	0.07	0.07	−0.01	**−0.15** ^*^	−0.07
7. BDI-II score	**−0.36** ^**^	**0.40** ^*^	0.12	**−0.37** ^*^	**−0.27** ^*^	0.12	0.12	**−0.21** ^*^	−0.10	−0.13
8. CGI-score	**−0.14** ^*^	**0.16** ^*^	0.08	**−0.17** ^*^	**−0.18** ^*^	0.03	0.03	−0.09	0.01	−0.11
9. Neurological disease^b^	0.07	0.07	−0.06	−0.01	−0.03	−0.02	−0.02	−0.12	0.04	0.07

ECQ, emotional competence questionnaire; BE, blame externalization; RN, rebellious nonconformity; SI, stress immunity; SP, social potency; CN, careless nonplanfulness; ME, machiavellian egocentricity; CH, coldheartedness. F, fearlessness; CogScore, cognitive composite score. Sex = Female (=1) and male (= 2) participants. BDI-II score, Beck Depression Inventory II score; CGI score, Clinical Global Impression Scale. ^*^ *p* < .05. ^**^p < .01. Benjamini-Yekutieli adjustments for all *p* values. Significant results are printed in bold.

a Spearman correlation analysis.

b 0 = none, 1 = present.

Statistical details on bivariate Pearson correlations between the study variables and their subscales, as well as means and standard deviations, are provided in Table 4. Since sex was significantly correlated with the ECQ score and the composite score, we additionally stratified the analysis for sex.

**Table 4:** Correlations, means, and standard deviations between study variables.

Variable		1	2	3	4	5	6	7	8	9	10	11	12	13	14	15	16	17	*M*	*SD*
PPI-R	1. BE	1	**0.27** ^***^	**−0.21** ^***^	**−0.18** ^***^	−0.11	**0.24** ^***^	**0.14** ^*^	0.10	**−0.33** ^***^	−0.12	**−0.26** ^***^	**−0.18** ^***^	**−0.30** ^***^	−0.13	−0.09	−0.04	−0.10	32.27	8.65
	2. RN		1	**0.16** ^*^	**0.27** ^***^	0.08	**0.44** ^***^	**0.29** ^***^	**0.42** ^***^	−0.08	0.04	−0.03	0.07	0.01	−0.04	0.01	−0.05	−0.03	48.60	10.89
	3. SI			1	**0.37** ^***^	**−0.17** ^**^	**−0.17** ^**^	**0.26** ^***^	**0.33** ^***^	**0.36** ^***^	0.07	**0.68** ^***^	**0.14** ^**^	**0.40** ^***^	0.06	−0.00	−0.00	0.02	33.67	7.15
	4. SP				1	−0.09	**0.13** ^*^	**0.15** ^**^	**0.16** ^**^	**0.31** ^***^	**0.29** ^***^	**0.21** ^***^	**0.41** ^***^	**0.43** ^***^	0.10	0.05	0.02	0.07	38.11	8.50
	5. CN					1	**0.20** ^**^	**0.15** ^*^	−0.01	**−0.22** ^***^	−0.13	**−0.26** ^***^	0.08	**−0.16** ^*^	−0.08	−0.09	−0.09	−0.11	30.00	5.78
	6. ME						1	**0.20** ^***^	0.03	−0.07	−0.09	**−0.19** ^***^	0.04	−0.10	−0.01	0.01	−0.01	−0.00	34.92	5.55
	7. CH							1	**0.19** ^***^	0.07	**−0.31** ^***^	0.14	0.02	−0.04	−0.04	−0.13	−0.06	−0.09	27.93	5.77
	8. F								1	0.04	−0.09	0.12	−0.02	0.01	0.01	−0.06	−0.03	−0.03	14.13	5.08
ECQ	9. RUE									1	**0.44** ^***^	**0.47** ^***^	**0.49** ^***^	**0.82** ^***^	**0.17** ^**^	0.12	0.11	**0.16** ^**^	3.11	0.57
	10. REO										1	0.14	**0.41** ^***^	**0.71** ^***^	0.10	**0.15** ^*^	0.04	0.12	3.58	0.68
	11. RCE											1	**0.15** ^*^	**0.56** ^***^	0.00	0.02	0.02	0.02	2.79	0.55
	12. EE												1	**0.77** ^***^	**0.19** ^***^	0.05	0.06	0.12	2.70	0.77
	13. ECQ Score													1	**0.17** ^**^	0.12	0.08	**0.15** ^*^	3.05	0.46
Cognitive domains	14. A/PS														1	**0.39** ^***^	**0.74** ^***^	**0.88** ^***^	0.02	1.03
	15. EF															1	**0.37** ^***^	**0.71** ^***^	0.10	0.99
	16. VL/M																1	**0.87** ^***^	3.05	0.96
	17. CogScore																	1	0.15	2.43

PPI-R, Psychopathic Personality Inventory-Revised; ECQ, emotional competence questionnaire; BE, blame externalization; RN, rebellious nonconformity; SI, stress immunity; SP, social potency; CN, careless nonplanfulness; ME, machiavellian egocentricity; CH, coldheartedness; F, fearlessness; RUE, recognizing and understanding emotions; REO, recognizing emotions of others; RCE, regulation and control of own emotions; EE, emotional expressivity; A/PS, attention/psychomotor speed; EF, executive function; VL/M, verbal learning/memory; M, mean; SD, standard deviation. ^*^p < .05; ^**^p < .01; and ^***^p < .001. Benjamini-Yekutieli adjustments for all *p* values. Significant results are printed in bold. Pearson correlation analyses results between study variables indicating significant relationships between the Emotional Competence Score (ECQ Score) and the Cognitive Composite Score (CogScore).

The ECQ score and the composite score were positively correlated, as well as the ECQ score and the PPT “Stress Immunity” and “Social Potency,” respectively. There was a significant negative relationship between the ECQ score and the PPT “Blame Externalisation” and “Careless Nonplanfulness,” respectively. Moreover, there were no significant relationships between the composite score and the PPT. When stratifying for sex, the relationship between the ECQ score and the composite score was not significant, neither in the female (*r* = 0.11, *p* = .106) nor in the male sample (*r* = 0.09, *p* = .280). Among female participants, there was a significant negative relationship between the ECQ score and the PPT “Blame Externalisation,” and “Careless Nonplanfulness.” Further, there was a significant positive association between the ECQ score and the PPT “Stress Immunity” and “Social Potency.” Among male participants, there was a significant negative relationship between the ECQ score and the PPT “Blame Externalisation,” and “Machiavellian Egocentricity.” Further, there was a significant positive association between the ECQ score and the PPT “Stress Immunity” and “Social Potency.” For both females and males, there were no significant relationships between the investigated cognitive functions and the PPT (see Supplementary Tables 4A,B in the Supplementary material for exact results).

### 3.3. Moderation analyses

To examine whether the relationship between emotional competence (ECQ score) and cognitive function (composite score) is moderated by the single PPT, we conducted eight moderation analyses. We found three outliers deviating more than three standard deviations, however, all Cook’s Distance values were below the critical threshold of 1, hence we included these cases in all further analyses. Results indicated that “Stress Immunity” (*R*^2^ = 0.014) and “Social Potency” (*R*^2^ = 0.011) significantly moderated the relationship between the ECQ score and the composite score. Similar *R*^2^ values were previously found in studies of cognition or emotion, and depression using moderation analyses (86, 87). Detailed moderation statistics depicting the unstandardized beta-weights, their standard errors, and the lower and upper bounds of the confidence intervals of main and interaction effects can be found in Table 5.

**Table 5:** Moderation analyses results for psychopathic personality traits scores moderating the relationship between emotional competence (ECQ Score) and cognitive composite score.

Effect	Estimate	*SE*	*t*	95% CI		*p*
				*LL*	*UL*	
Blame externalization (BE)						
Constant	0.16	0.13	1.20	−0.10	0.40	.230
ECQ score	**0.67** ^*^	0.28	2.37	0.13	1.23	.018
BE	−0.02	0.02	−1.22	−0.05	0.01	.222
ECQ score × BE	−0.04	0.03	−1.46	−0.10	0.01	.145
Rebellious nonconformity (RN)						
Constant	0.21	0.13	1.65	−0.04	0.45	.100
ECQ score	**0.78** ^**^	0.27	2.88	0.26	1.32	.004
RN	−0.01	0.01	−0.54	−0.03	0.02	.588
ECQ score × RN	0.01	0.03	0.41	−0.03	0.06	.679
Stress immunity (SI)						
Constant	0.10	0.14	0.74	−0.17	0.36	.462
ECQ score	**0.87** ^**^	0.28	2.95	0.31	1.42	.003
SI	−0.02	0.02	−1.10	−0.06	0.02	.273
ECQ score × SI	**0.08** ^*^	0.04	2.29	0.01	0.15	.023
Social potency (SP)						
Constant	0.11	0.14	0.78	−0.17	0.37	.434
ECQ score	**0.77** ^*^	0.30	2.55	0.19	1.36	.011
SP	−0.00	0.02	−0.13	−0.03	0.03	.898
ECQ score × SP	**0.06** ^*^	0.03	2.01	−0.00	0.12	.045
Careless nonplanfulness (CN)						
Constant	0.21	0.13	1.64	−0.04	0.46	.103
ECQ score	**0.71** ^*^	0.28	2.57	0.18	1.27	.011
CN	−0.04	0.02	−1.58	−0.08	0.01	.116
ECQ score × CN	−0.02	0.05	−0.04	−0.10	0.10	.967
Machiavellian egocentricity (ME)						
Constant	0.22	0.13	1.76	−0.03	0.47	.080
ECQ score	**0.77** ^**^	0.27	2.85	0.27	1.30	.005
ME	0.01	0.02	0.23	−0.04	0.05	.822
ECQ score × ME	0.06	0.05	1.18	−0.05	0.15	.240
Coldheartedness (CH)						
Constant	0.21	0.13	1.64	−0.04	0.45	.102
ECQ score	**0.78** ^**^	0.27	2.87	0.26	1.31	.004
CH	−0.03	0.02	−1.67	−0.08	0.01	.095
ECQ score × CH	−0.02	0.05	−0.54	−0.12	0.08	.590
Fearlessness (F)						
Constant	0.21	0.13	1.65	−0.04	0.45	.099
ECQ score	**0.79** ^**^	0.27	2.89	0.27	1.32	.004
*F*	−0.02	0.02	−0.67	−0.06	0.03	.503
ECQ score × F	−0.06	0.06	−0.12	−0.12	0.11	.904

Estimate, unstandardized regression weight; SE, standard error of the unstandardized regression weight; 95% CI, 95%—confidence intervals based on 10,000 samples (BCa bootstrapping); LL, lower level of the 95% confidence interval; UL, upper level of the 95% confidence interval; ^*^p < .05, ^**^p < .01. Significant results are printed in bold. Results indicate a significant interaction effect between the ECQ score and the cognitive composite score, which is positively moderated by SI and SP.

In an additional analysis, we conducted all moderation analyses without the three outliers. These analyses indicate the same results as the analyses including the outliers [i.e., significantly moderating effects of “Stress Immunity” (*R*^2^ = 0.034) and “Social Potency” (*R*^2^ = 0.031)]. Detailed results can be found in the Supplementary Table 5A.

**Figure 1:**
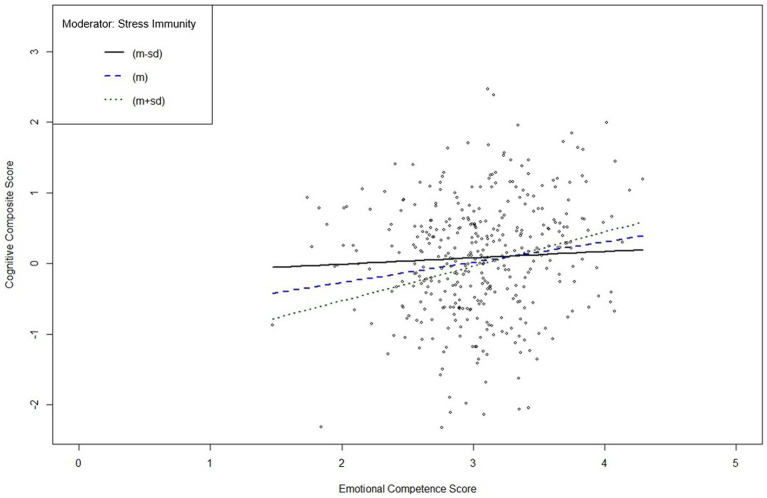
Interaction plot of the simple slope analysis of psychopathic personality trait “Stress Immunity” in individuals with depression. *m*, mean; *sd*, standard deviation. Simple slope analysis of the moderating effect of “Stress Immunity” on the relationship between the Emotional Competence Score and the Cognitive Composite Score indicates a significant moderation effect at high (+1 *sd* above *m*; solid black line) and low (−1 *sd* below *m*; dotted black line) values of “Stress Immunity” (*m* is represented by the blue dashed line). Subsequent significance tests indicate a significant positive moderating effect for individuals with depression and high values in “Stress Immunity,” but not for individuals with depression and low values in “Stress Immunity.”

To further examine the significant interaction effects, we conducted simple slope analyses. Figure 1 reveals a plot indicating that the ECQ score significantly predicted the composite score at high and low values of the moderator “Stress Immunity.” Further examination indicated that in individuals with depression low in the PPT “Stress Immunity” there was no statistically significant relationship between emotional competence and cognitive functions (*b* = 0.27, *t* = 0.69, *p* = .492), but in individuals high in “Stress Immunity” there was a significantly positive relationship (*b* = 1.46, *t* = 3.72, *p* < .001).

**Figure 2:**
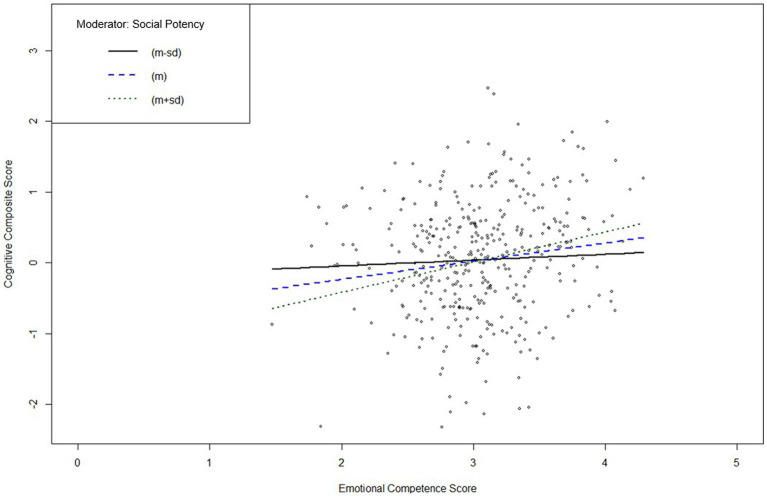
Interaction plot of the simple slope analysis of psychopathic personality trait “Social Potency” in individuals with depression. *m*, mean; *sd*, standard deviation. Simple slope analysis of the moderating effect of “Social Potency” on the relationship between the Emotional Competence Score and the Cognitive Composite Score indicates a significant moderation effect at high (+1 *sd* above *m*; solid black line) and low (−1 *sd* below *m*; dotted black line) values of “Social Potency” (*m* is represented by the blue dashed line). Subsequent significance tests indicate a significant positive moderating effect for individuals with depression and high values in “Social Potency,” but not for individuals with depression and low values in “Social Potency.”

Figure 2 shows that the ECQ score significantly predicted the composite score at high and low values of the moderator “Social Potency.” The analysis indicated that, in depression, there was no statistically significant relationship between emotional competence and cognitive functions in individuals with lower scores in the PPT “Social Potency” (*b* = 0.25, *t* = 0.62, *p* = .535), but there was a statistically significant relationship between emotional competence and cognitive functions in individuals high in “Social Potency” (*b* = 1.28, *t* = 3.28, *p* < .01).

## 4. Discussion

This study aimed to investigate the relationship between PPT, emotional competence, and cognitive functions in individuals with depression. We sought to find out whether there is a significant association between emotional competence and cognitive functions, and whether PPT are significantly related to emotional competence and cognitive functions in individuals with depression. Further, the current study investigated whether PPT have a moderating role in the relationship between emotional competence and cognitive functions. Our results indicate that there is a significant positive relationship between emotional competence and cognitive functions in individuals with depression, indicating that higher emotional competence is related to better cognitive abilities in the domains attention, executive function, and verbal learning/memory. When stratifying the results for sex, the relationship between emotional competence and cognitive functions did not remain significant, neither in the female nor in the male sub-sample. Moreover, we found that some of the PPT are positively, and some are negatively related to emotional competence, but not related to any of the investigated cognitive functions. This was found for both the general and the stratified sample. Last, we found that the PPT “Stress Immunity” and “Social Potency” significant positively moderated the relationship between emotional competence and cognitive functions in individuals with depression. This finding indicates that being immune toward stress and anxiety (i.e., “Stress Immunity”) and being superficially charming and dominant toward others (i.e., “Social Potency”; 75) might serve as strengthening and protective traits in the relationship between emotional competence and cognitive functions in individuals with depression. Interestingly, none of the other PPT had a significant influence on the relationship between emotional competence and cognitive functions.

Our result of a positive relationship between emotional competence and cognitive functions in individuals with depression is in line with our hypothesis and is supported by existing literature (e.g., 88, 89). We strengthen the already existing work by highlighting the importance of examining emotional and cognitive abilities together when investigating mood disorders, which was also previously proposed (90). Looking closely at our results, it can be observed that the subcomponents of emotional competence were related to the cognitive function of attention/processing speed, but not the other domains. This poses an interesting finding, since previous research mainly found evidence on a negative link between emotional competence and executive dysfunction in depression (e.g., 91). In addition to these previous findings, our results support studies which show that emotional aspects and attention are positively related (e.g., 92). When stratifying the results according to sex, the relationship between emotional competence and cognitive functions did not remain significant, neither in among female nor male participants. For the moderation analyses, we refrained from including sex as a covariate due to the possibility of reduced availability of degrees of freedom, statistical power, and amount of explainable variance in the outcomes (93). Moreover, in line with current recommendations on the inclusion of control variables (94), it was aimed not to force the relationship between emotional competence and cognitive functions to be the same across sex, thus resulting in not including sex as a control variable.

Moreover, we found that the PPT “Blame Externalisation” and “Careless Nonplanfulness” were significantly negatively associated with emotional competence. This finding aligns previous studies stating that PPT are associated with both emotional dysregulation and externalization behavior, because individuals high in PPT are more likely to blame external factors for their behavior rather than confronting their emotions (95, 96). Other studies revealed that the superior factor of both PPT subscales, namely “Self-Centered Impulsivity,” which assesses a narcissistic and reckless tendency to exploit and blame others, is associated with a deficit in self-conscious emotions and adopting other’s perspective (97) and is thus seen as maladaptive behavior (98). Indeed, our findings show that both PPT are negatively related to the ECQ subscale “Recognizing and Understanding Emotions of Others.” Contrary, the PPT “Stress Immunity” and “Social Potency” were significantly positively related to emotional competence, which is in line with other studies revealing a negative association between stress immunity and emotional dysregulation (89) or a positive association between emotional intelligence and both traits’ superior factor “Fearless Dominance,” which comprises physical and social boldness as well as immunity to anxiety. This relationship is referred to as “successful psychopathy” and is thus largely associated with adaptive behavior (98, 99). This finding can be explained by the fact that psychopathic stress immunity helps to maintain a healthy emotional distance (97) and can thus be seen as a key element in emotional competence. “Social Potency,” on the other hand, is a PPT that circumcises the influential and manipulative behavior, as well as the deceptive use of emotions in social situations. This requires at least some understanding of one’s own and other’s emotions, thus possibly leading to the positive association with emotional competence, although it is discussed that this trait just reflects the impression but not a real presence of emotional competence (100). Interestingly, we did not find any significant associations between PPT and cognitive domains or overall cognition score, thus only partly confirming our second hypothesis. This finding is contrary to studies revealing major cognitive deficits in PPT (31, 101), specifically when it comes to attention and cognitive control (27, 102, 103). Moreover, our result of no significant association between PPT and cognitive functions is surprising since we investigated individuals with depression whose cognitive functions have also previously been shown to be impaired in general (e.g., 104). Possibly, the interaction between cognitive functions and PPT is different in those individuals than in other populations; however, further research is needed to disentangle the underlying mechanisms.

Finally, we found that only the PPT “Stress Immunity” and “Social Potency” are able to significant positively moderate the relationship between emotional competence and cognitive functions, thus partly supporting our hypothesis. Another study examining the moderating role of PPT in the relationship between cognitive functions and emotion processing, as measured by response to threatening stimuli, in mentally healthy individuals revealed that the superior factor of “Social Potency” and “Stress Immunity,” namely “Fearless Dominance” interacts significant positively with cognitive and affective processing (105). Taken together with our finding of positive moderating effects of “Stress Immunity” and “Social Potency,” our study results also support the findings of Dalkner and colleagues (34), who concluded that there may be a protective function of PPT in depression. Thus, being more resilient to potentially anxiety-inducing events and showing more charming and interpersonally bold behavior seems to have a positive effect on the interaction between emotional competence and cognitive functions in depression. This finding is also in line with the fact that stressful environments (106, 107) can result in the manifestation of depressive symptoms and that individuals with depression tend to be shyer and more insecure in social contexts (108), often showing symptomatic social withdrawal. Our result regarding the positive influence of “Social Potency” and “Stress Immunity” thus implies that stress coping strategies and training of social skills should be targeted in the treatment of depressive symptomatology. All other PPT did not significantly moderate the relationship between emotional competence and cognitive functions. This poses an interesting finding, since psychopathy is connected to both emotional and cognitive aspects (e.g., 48). Possibly, the interaction between emotional competence and cognitive functions in depression works independent of most PPT or is influenced by other aspects (e.g., symptom severity). Future studies should look more closely at these relationships to determine the influence of PPT on these constructs, consider possibly other influencing variables, and transfer this knowledge into everyday treatment of depressive symptoms. For example, since depression and PPT share underlying neurobiological processes, further research could examine both constructs with regard to their physiological correlates. Ultimately, novel findings on personality traits in depression could be integrated in the therapeutic process, thus supporting the multifactorial treatment of psychiatric disorders in general.

### 4.1. Limitations

The study results should be interpreted with following limitations in mind. First, we only used self-report measures, which has to be observed critically when examining PPT due to a possible social desirability bias (109). It should also be noted that the PPT scores of this specific sample were located in the lower area of the total possible scale scores. Secondly, no data of a healthy control group were collected, thus no comparisons can be made and we do not know whether our results are limited to depressive subjects. Future studies should include a control group to fully understand the investigated relationships. Third, we did not control for socio-demographic (e.g., sex, education) or illness-related variables such as number of depressive episodes (first-time or recurrent) or illness duration, which could possibly influence our findings. Fourth, the current study did not include imaging or molecular data, thus only depicting subjectively measured findings. Nevertheless, our results serve as a stepping stone for further examinations including imaging or molecular methods to determine the relationships between cognition, emotion, and PPT. Finally, due to the correlative and cross-sectional study design, no statements about the causal relationships between the examined constructs can be made. In light of our results, however, the importance of looking more closely at the mechanisms driving these relationships is given and should be considered in future studies.

### 4.2. Conclusion

This study reveals a positive relationship between emotional competence and cognitive functions (including attention/psychomotor speed, executive functions, and verbal learning/memory) in individuals with depression. Further, it was observed that single PPT, but not all, are related to emotional competence. In particular, the PPT “Stress Immunity” and “Social Potency” positively moderated the relationship between emotional competence and cognitive functions, thus indicating the importance of developing stress coping mechanisms and better social skills in individuals with depression to maintain a good interaction between emotional competence and cognitive functions. Future studies should aim to examine the relationships between PPT, emotion, and cognitive functions in individuals with psychiatric disorders more closely, as considering personality traits in research can possibly improve the overall treatment outcome and help understand the mechanisms of such diseases. Moreover, the integration of PPT in the treatment process could enrich the therapeutic possibilities by identifying individual traits as resilience-strengthening or potentially harmful factors for depressive symptomatology. Subsequently, measures preventing depressive deterioration in cognitive and emotional domains could be established. In the interest of translational research, these results should encourage the consideration of PPT in the clinical treatment of several psychiatric disorders.

## Data availability statement

The datasets presented in this study can be found in online repositories. The names of the repository/repositories and accession number(s) can be found at: https://doi.org/10.17605/OSF.IO/5GUD9, OSF repository.

## Ethics statement

The studies involving human participants were reviewed and approved by Ethics committee of the Federal State Upper Austria (EK-number: E-24-14). The patients/participants provided their written informed consent to participate in this study.

## Author contributions

ES and ND: conceptualization, formal analysis, and methodology. ND, KS, ER, and BR: data curation. ES: funding acquisition, software, visualization, and writing—original draft. ND: investigation and supervision. ND, ER, and BR: project administration and resources. ES, ND, KS, ER, and BR: validation and writing—review and editing. All authors contributed to the article and approved the submitted version.

## Funding

The work was supported by the Open Access publication costs are funded by the funding program of the Province of Styria, Austria (ABT12-632902/2022-7).

## Conflict of interest

The authors declare that the research was conducted in the absence of any commercial or financial relationships that could be construed as a potential conflict of interest.

## Publisher’s note

All claims expressed in this article are solely those of the authors and do not necessarily represent those of their affiliated organizations, or those of the publisher, the editors and the reviewers. Any product that may be evaluated in this article, or claim that may be made by its manufacturer, is not guaranteed or endorsed by the publisher.

## References

[1] Otte C Gold SM Penninx BW Pariante CM Etkin A Fava M. Major depressive disorder. Nat Rev Dis Primers. (2016) 2:16065. doi: 10.1038/nrdp.2016.65 27629598

[2] Hill KE South SC Egan RP Foti P. Abnormal emotional reactivity in depression: contrasting theoretical models using neurophysiological data. Biol Psychol. (2019) 141:35–43. doi: 10.1016/j.biopsycho.2018.12.011, PMID: 30597188

[3] Joorman J Stanton CH. Examining emotion regulation in depression: a review and future directions. Behav Res Ther. (2016) 86:35–49. doi: 10.1016/j.brat.2016.07.007, PMID: 27492851

[4] Visted E Vollestad J Nielsen MB Schanche E. Emotion regulation in current and remitted depression: a systematic review and meta-analysis. Front Psychol. (2018) 9:756. doi: 10.3389/fpsyg.2018.00756, PMID: 29867700 PMC5968125

[5] Bourke C Douglas K Porter R. Processing of facial emotion expression in major depression: a review. Aust N Z J Psychiatry. (2010) 44:681–96. doi: 10.3109/00048674.2010.496359, PMID: 20636189

[6] Rodman AM Jenness JL Weissman DG Pine DS McLaughlin KA. Neurobiological markers of resilience to depression following childhood maltreatment: the role of neural circuits supporting the cognitive control of emotion. Biol Psychiatry. (2019) 86:464–73. doi: 10.1016/j.biopsych.2019.04.033, PMID: 31292066 PMC6717020

[7] Rock PL Roiser JP Riedel WJ Blackwell AD. Cognitive impairment in depression: a systematic review and meta-analysis. Psychol Med. (2013) 44:2029–40. doi: 10.1017/S0033291713002535 24168753

[8] Roca M Vives M López-Navarro E García-Campayo J Gili M. Cognitive impairments and depression: a critical review. Actas Esp Psiquitr. (2015) 43:5. 26320897

[9] Semkowska M Quinlivan L O’Grady T Johnson R Collins A O’Connor J. Cognitive function following a major depressive episode: a systematic review and meta-analysis. Lancet Psychiatry. (2019) 6:851–61. doi: 10.1016/S2215-0366(19)30291-3, PMID: 31422920

[10] Hakulinen C Elovainio M Pulkki-Råback L Virtanen M Kivimäki M Jokela M. Personality and depressive symptoms: individual participant meta-analysis of 10 cohort studies. Depress Anxiety. (2015) 32:461–70. doi: 10.1002/da.22376, PMID: 26014798 PMC4605994

[11] Hare RD. Psychopathy: a clinical construct whose time has come. Crim Justice Behav. (1996) 23:25–54. doi: 10.1177/0093854896023001004

[12] Hyde L Byrd A Votruba-Drzal E Hariri A Manuck S. Amygdala reactivity and negative emotionality: divergent correlates of antisocial personality and psychopathy traits in a community sample. J Abnorm Psychol. (2014) 123:214–24. doi: 10.1037/a0035467, PMID: 24661171 PMC4008968

[13] Blackburn R. Personality disorder and antisocial deviance: comments on the debate on the structure of the psychopathy checklist—revised. J Personal Disord. (2007) 21:142–59. doi: 10.1521/pedi.2007.21.2.142, PMID: 17492918

[14] Abdalla-Filho E Völlm B. Does every psychopath have an antisocial personality disorder? Braz J Psychiatry. (2020) 42:241–2. doi: 10.1590/1516-4446-2019-0762, PMID: 32074231 PMC7236162

[15] Casey H Rogers RD Burns T Yiend J. Emotion regulation in psychopathy. Biol Psychol. (2013) 92:541–8. doi: 10.1016/j.biopsycho.2012.06.011 23079384

[16] Garofalo C Neumann CS Kosson DS Velotti P. Psychopathy and emotion dysregulation: more than meets the eye. Psychiatry Res. (2020) 290:113160. doi: 10.1016/j.psychres.2020.113160, PMID: 32526515

[17] Garofalo C Neumann CS Velotti P. Difficulties in emotion regulation and psychopathic traits in violent offenders. J Crime Justice. (2018) 57:116–25. doi: 10.1016/j.jcrimjus.2018.05.013

[18] Hussain Z Wegmann E Griffiths MD. The association between problematic social networking site use, dark triad traits, and emotion dysregulation. BMC Psychol. (2021) 9:160. doi: 10.1186/s40359-021-00668-6, PMID: 34663456 PMC8525015

[19] Newman JP Lorenz AR. Response Modulation and Emotion Processing: Implications for Psychopathy and Other Dysregulatory Psychopathology. Handbook of Affective Sciences. (2003) Oxford University Press, 904–929

[20] Cigna MH Guay JP Renaud P. Psychopathic traits and their relation to facial affect recognition. Pers Individ Differ. (2017) 117:210–5. doi: 10.1016/j.paid.2017.06.014

[21] Glenn AL Efferson LM Kastner RM Johnson AK Remmel RJ. Psychopathy and the perception of the genuineness of facial expressions. Pers Individ Differ. (2022) 187:111439. doi: 10.1016/j.paid.2021.111439

[22] Dawel A O’Kearney R McKone E Palermo R. Not just fear and sadness: meta-analytic evidence of pervasive emotion recognition deficits for facial and vocal expressions in psychopathy. Neurosci Biobehav Rev. (2012) 36:2288–304. doi: 10.1016/j.neubiorev.2012.08.006, PMID: 22944264

[23] Marsh AA Blair RJR. Deficits in facial affect recognition among antisocial populations: a meta-analysis. Neurosci Biobehav Rev. (2008) 32:454–65. doi: 10.1016/j.neubiorev.2007.08.003, PMID: 17915324 PMC2255599

[24] Künecke J Mokros A Olderbak S Wilhelm O. Facial responsiveness of psychopaths to the emotional expressions of others. PLoS One. (2018) 13:e0190714. doi: 10.1371/journal.pone.0190714, PMID: 29324826 PMC5764293

[25] Baliousis M Duggan C McCarthy L Huband N Völlm B. Executive function, attention, and memory deficits in antisocial personality disorder and psychopathy. J Psychiatr Res. (2019) 278:151–61. doi: 10.1016/j.psychres.2019.05.046, PMID: 31200194

[26] Hamilton RKB Baskin-Sommers AR Newman JP. Relation of frontal N100 to psychopathy-related differences in selective attention. Biol Psychol. (2014) 103:107–16. doi: 10.1016/j.iopsycho.2014.08.012 PMC4407830 25179538

[27] Sadeh N Verona E. Psychopathic personality traits associated with abnormal selective attention and impaired cognitive control. Neuropsychology. (2008) 22:669–80. doi: 10.1037/a0012692, PMID: 18763886 PMC2538613

[28] Vitale JE Brinkley CA Hiatt KD Newman JP. Abnormal selective attention in psychopathic female offenders. Neuropsychology. (2007) 21:301–12. doi: 10.1037/0894-4105.21.3.301, PMID: 17484593 PMC3136379

[29] Bagshaw R Gray NS Snowden RJ. Executive function in psychopathy: the tower of London, Brixton spatial anticipation and the Hayling sentence completion tests. Psychiatry Res. (2014) 220:483–9. doi: 10.1016/j.psychres.2014.07.031, PMID: 25110313

[30] Snowden RJ Gray NS Pugh S Atkinson G. Executive function as a function of sub-clinical psychopathy. Pers Individ Differ. (2013) 55:801–4. doi: 10.1016/j.paid.2013.06.016

[31] Kiehl KA. A cognitive neuroscience perspective on psychopathy: evidence for paralimbic system dysfunction. Psychiatry Res. (2006) 142:107–28. doi: 10.1016/j.psychres.2005.09.013, PMID: 16712954 PMC2765815

[32] Shih SI Chi NW Wu CC Wang SY. When dark meets blue: the relations between dark triad personality and depression symptoms. Curr Psychol. (2019) 40:6110–7. doi: 10.1007/s12144-019-00549-7

[33] Šram Z. The comorbidity of psychopathy and depression: across different ethnic and sex groups. Int J Indian Psychol. (2017) 4:3. doi: 10.25215/0403.051

[34] Dalkner N Reininghaus EZ Riedrich K Rieger A Birner A Fellendorf FT. Psychopathic personality factor “fearless dominance” is related to low self-reported stress-levels, fewer psychiatric symptoms, and more adaptive stress coping in psychiatric disorders. Psychiatry Res. (2018) 270:68–77. doi: 10.1016/j.psychres.2018.09.018, PMID: 30245379

[35] Hansen AL Stokkeland L Pallesen S Johnsen BH Waage L. The relationship between the psychopathy checklist-revised and the MMPI-2: a pilot study. Psychol Rep. (2013) 112:445–57. doi: 10.2466/03.09.PR0.112.2.445-457, PMID: 23833874

[36] Willemsen J Vanheule S Verhaeghe P. Psychopathy and lifetime experiences of depression. Crim Behav Ment Health. (2011) 21:279–94. doi: 10.1002/cbm.812, PMID: 21469239

[37] Battaglia S Harrison BJ Fullana MA. Does the human ventromedial prefrontal cortex support fear learning, fear extinction or both? A commentary on subregional contributions. Mol Psychiatry. (2022) 27:784–6. doi: 10.1038/s41380-021-01326-4, PMID: 34667263

[38] Battaglia S Cardellicchio P Di Fazio C Nazzi C Fracasso A. The influence of vicarious fear-learning in "infecting" reactive action inhibition. Front Behav Neurosci. (2022) 16:946263. doi: 10.3389/fnbeh.2022.946263, PMID: 35941933 PMC9355887

[39] Fullana MA Harrison BJ Soriano-Mas C Vervliet B Cardoner N Àvila-Parcet A. Neural signatures of human fear conditioning: an updated and extended meta-analysis of fMRI studies. Mol Psychiatry. (2015) 88:21. doi: 10.1038/mp.2015.88 26122585

[40] Battaglia S Cardellicchio P Di Fazio C Nazzi C Fracasso A Borgomaneri S. Stopping in (e)motion: reactive action inhibition when facing valence-independent emotional stimuli. Front Behav Neurosci. (2022) 16:998714. doi: 10.3389/fnbeh.2022.998714, PMID: 36248028 PMC9561776

[41] Frodl T Schaub A Banac S Charypar M Jäger M Kümmler P. Reduced hippocampal volume correlates with executive dysfunctioning in major depression. J Psychiatry Neurosci. (2006) 31:316–23. PMID: 16951734 PMC1557684

[42] Sheline YI Gado MH Kraemer HC. Untreated depression and hippocampal volume loss. Am J Psychiatry. (2003) 160:8. doi: 10.1176/appi.ajp.160.6.1516 12900317

[43] Lange C Irle E. Enlarged amygdala volume and reduced hippocampal volume in young women with major depression. Psychol Med. (2004) 34:1059–64. doi: 10.1017/s0033291703001806, PMID: 15554576

[44] Weniger G Lange C Irle E. Abnormal size of the amygdala predicts impaired emotional memory in major depressive disorder. J Affect Disord. (2006) 94:219–29. doi: 10.1016/j.jad.2006.04.017, PMID: 16740316

[45] Pizzagalli DA Roberts AC. Prefrontal cortex and depression. Neuropsychopharmacology. (2022) 47:225–46. doi: 10.1038/s41386-021-01101-7, PMID: 34341498 PMC8617037

[46] Van Tol M-J van der Wee NJA van den Heuvel OA Nielen MMA Dmenescu LR Aleman A. Regional brain volume in depression and anxiety disorders. Arch Gen Psychiatry. (2010) 67:10. doi: 10.1001/archgenpsychiatry.2010.121 20921116

[47] Salzman D Fusi S. Emotion, cognition, and mental state representation in amygdala and prefrontal cortex. Annu Rev Neurosci. (2010) 33:173–202. doi: 10.1146/annurev.neuro.051508.135256, PMID: 20331363 PMC3108339

[48] Anderson NE Steele VR Maurer JM Rao V Koenigs MR Decety J. Differentiating emotional processing and attention in psychopathy with functional neuroimaging. Cogn Affect Behav Neurosci. (2017) 17:491–515. doi: 10.3758/s13415-016-0493-5, PMID: 28092055 PMC5404945

[49] Sommer M Hajak G Döhnel K Schwerdtner J Meinhardt J Müller JL. Integration of emotion and cognition in patients with psychopathy. Prog Brain Res. (2006) 156:1994. doi: 10.1016/S0079-6123(06)56025-X, PMID: 17015096

[50] Weber S Habel U Amunts K Schneider F. Structural brain abnormalities in psychopaths–a review. Behav Sci Law. (2008) 26:7–28. doi: 10.1002/bsl.802, PMID: 18327824

[51] Miglin R Rodriguez S Bounoua N Sadeh N. A multidimensional examination of psychopathy traits and gray matter volume in adults. Soc Cogn Affect Neurosci. (2022) 17:662–72. doi: 10.1093/scan/nsab131, PMID: 34878140 PMC9250300

[52] Johanson M Vaurio O Tiihonen J Lähteenvuo M. A systematic literature review of neuroimaging of psychopathic traits. Front Psychol. (2020) 10:1027. doi: 10.3389/fpsyt.2019.01027, PMID: 32116828 PMC7016047

[53] Frazier A Ferreira PA Gonzales JE. Born this way? A review of neurobiological and environmental evidence for the etiology of psychopathy. Pers Neurosci. (2019) 2:e8. doi: 10.1017/pen.2019.7, PMID: 32435743 PMC7219694

[54] Palumbo S Mariotti V Vellucci S Antonelli K Anderson N Harenski C. HTR1B genotype and psychopathy: Main effect and interaction with paternal maltreatment. Psychoneuroendocrinology. (2022) 144:105861. doi: 10.1016/j.psyneuen.2022.105861, PMID: 35853382

[55] Kanen JW Arntz FE Yellowlees R Cardinal RN Price A Christmas DM. Serotonin depletion amplifies distinct human social emotions as a function of individual differences in personality. Transl Psychiatry. (2021) 11:81. doi: 10.1038/s41398-020-00880-9, PMID: 33518708 PMC7847998

[56] Cowen PJ Browning M. What has serotonin to do with depression? World Psychiatry. (2015) 14:158–60. doi: 10.1002/wps.20229, PMID: 26043325 PMC4471964

[57] Glenn AL Raine A. The neurobiology of psychopathy. Psychiatr Clin North Am. (2008) 31:463–75. doi: 10.1016/j.psc.2008.03.004 18638646

[58] Van Honk J Schutter DJLG. Unmasking feigned sanitiy: a neurobiological model of emotion processing in primary psychopathy. Cogn Neuropsychiatry. (2006) 11:285–306. doi: 10.1080/13546800500233728, PMID: 17354073

[59] Hinkelmann K Moritz S Botzenhardt J Riedesel K Wiedemann K Kellner M. Cognitive impairment in major depression: association with salivary cortisol. Biol Psychiatry. (2009) 66:879–85. doi: 10.1016/j.biopsych.2009.06.023, PMID: 19709646

[60] Rivera-Bonet CN Birn RM Ladd CO Meyerand ME Abercrombie HC. Cortisol effects on brain functional connectivity during emotion processing in women with depression. J Affect Disord. (2021) 287:247–54. doi: 10.1016/j.jad.2021.03.034, PMID: 33799044 PMC8128282

[61] Osumi T Shimazaki H Imai A Sugiura Y Ohira H. Psychopathic traits and cardiovascular responses to emotional stimuli. Pers Individ Differ. (2007) 42:1391–402. doi: 10.1016/j.paid.2006.10.016

[62] Hu MX Lamers F de Geus EJC Penninx BWJH. Differential autonomic nervous system reactivity in depression and anxiety during stress depending on type of stressor. Psychosom Med. (2016) 78:562–72. doi: 10.1097/PSY.0000000000000313, PMID: 26910796

[63] Koudys JW Traynor JM Rodrigo AH Carcone D Ruocco AC. The NIMH research domain criteria (RDoC) initiative and its implications for research on personality disorder. Curr Psychiatry Rep. (2019) 21:37. doi: 10.1007/s11920-019-1023-2, PMID: 31030293

[64] Etkin P De Caluwé E Ibánez MI Ortet G Mezquita L. Personality development and its associations with the bifactor model in psychopathology in adolescence. J Res Pers. (2022) 97:104205. doi: 10.1016/j.jrp.2022.104205

[65] Sellbom M Carragher N Sunderland M Calear AL Batterham PJ. The role of maladaptive personality domains across multiple levels of the HiTOP structure. Personal Ment Health. (2019) 14:30–50. doi: 10.1002/pmh.1461, PMID: 31397079

[66] Widiger TA Sellbom M Chmielewski M Clark LA DeYoung CG Kotov R. Personality in a hierarchical model of psychopathology. Clin Psychol Sci. (2019) 7:77–92. doi: 10.1177/2167702618797105

[67] Ramey T Regier PS. Cognitive impairment in substance use disorders. CNS Spectr. (2019) 24:102–13. doi: 10.1017/S1092852918001426, PMID: 30591083 PMC6599555

[68] Faul F Erdfelder E Lang AG Buchner A. G^*^power 3: a flexible statistical power analysis program for the social, behavioral and biomedical sciences. Behav Res Methods. (2007) 39:175–91. doi: 10.3758/BF03193146 17695343

[69] Reininghaus B Riedrich K Dalkner N Lehner LA Rieger A Hamm C. Physical health in individuals with psychiatric disorders in Austria. J Affect Disord. (2019) 257:38–44. doi: 10.1016/j.jad.2019.06.045 31299403

[70] Reininghaus EZ Dalkner N Riedrich K Fuchs D Gostner JM Reininghaus B. Sex specific changes in tryptophan breakdown over a 6 week treatment period. Front Psychol. (2019) 10:74. doi: 10.3389/fpsyt.2019.0007460, PMID: 30846946 PMC6393336

[71] Kummer S Dalkner N Schwerdtfeger A Hamm C Schwalsberger K Reininghaus B. The conscientiousness-health link in depression: results from a path analysis. J Affect Disord. (2021) 295:1220–8. doi: 10.1016/j.jad.2021.09.017, PMID: 34706436

[72] Rindermann H. Emotionale-Kompetenz-Fragebogen: EKF [Emotional Competence Questionnaire]. Göttingen: Hogrefe (2009).

[73] Alpers GW Eisenbarth H. Psychopathic Personality Inventory Revised (PPI-R) Manual–German Version. Göttingen: Hogrefe (2008).

[74] Lilienfeld SO Widows MR. Psychopathic Personality Inventory–Revised: Professional Manual. Lutz, FL: Psychological Assessment Resources (2005).

[75] Tonnaer F Cima M Sijtsma K Uzieblo K Lilienfeld SO. Screening for psychopathy: validation of the psychopathic personality inventory-short form with reference scores. J Psychopathol Behav Assess. (2012) 35:153–61. doi: 10.1007/s10862-012-9333-2

[76] Reitan RM. Trail Making Test: Manual for Administration, Scoring, and Interpretation. Indianapolis, IN: Indiana University Press (1956).

[77] Bäumler G. Farbe-Wort-Interferenztest (FWIT) nach J.R Stroop: Handanweisung [Colour-Word-Interference Test by J.R. Stroop: Manual]. Göttingen: Hogrefe (1985).

[78] Brickenkamp R Schmidt-Atzert L Liepmann D. Test d2-Revision: Aufmerksamkeits- und Konzentrationstest [Test d2-Revision: Attention- and Concentration Test]. Göttingen: Hogrefe (2010).

[79] Niemann H Sturm W Thöne-Otto AIT Willmes K. CVLT California Verbal Learning Test. German Adaptation. Manual. Frankfurt am Main. Germany: Pearson Assessment (2008).

[80] Wechsler D. WAIS-IV Administration and Scoring Manual. San Antonio, TX: The Psychological Corporation (2008).

[81] Beck AT Ward CH Mendelson M Mock J Erbaugh J. An inventory for measuring depression. Arch Gen Psychiatry. (1961) 4:561–71. doi: 10.1001/archpsyc.1961.01710120031004 13688369

[82] Guy W. Clinical global impressions In: Guy W, editor. ECDEU Assessment Manual for Psychopharmacology: Publication ADM. Rockville, MD: National Institute of Mental Health, Psychopharmacology Research Branch (1976). 76–338.

[83] Hasan A Guse B Cordes J Wölwer W Winterer G Gaebel W. Cognitive effects of high-frequency rTMS in schizophrenia patients with predominant negative symptoms: results from a multicenter randomized sham-controlled trial. Schizophr Bull. (2016) 42:608–18. doi: 10.1093/schbul/sbv142, PMID: 26433217 PMC4838079

[84] Benjamini Y Yekutieli D. The control of the false discovery rate in multiple testing under dependency. Ann Stat. (2001) 29:4. doi: 10.1214/aos/1013699998

[85] Hayes AF. The PROCESS macro for SPSS, SAS, and R. (2021). Available at: processmacro.org/download.html

[86] Duarte AC Matos AP Marques C. Cognitive emotion regulation strategies and depressive symptoms: gender’s moderating effect. Procedia Soc Behav Sci. (2015) 165:275–83. doi: 10.1016/j.sbspro.2014.12.632

[87] Gilbert BD Christopher MS. Mindfulness-based attention as a moderator of the relationship between depressive affect and negative cognitions. Cogn Ther Res. (2010) 34:514–21. doi: 10.1007/s10608-009-9282-6

[88] Joormann J Gotlib IH. Emotion regulation in depression: relation to cognitive inhibition. Cognit Emot. (2010) 24:281–98. doi: 10.1080/02699930903407948, PMID: 20300538 PMC2839199

[89] Joormann J Vanderlind MW. Emotion regulation in depression: the role of biased cognition and reduced cognitive control. Clin Psychol Sci. (2014) 2:402–21. doi: 10.1177/2167702614536163

[90] Lima IMM Peckham AD Johnson SL. Cognitive deficits in bipolar disorders: implications for emotion. Clin Psychol Rev. (2018) 59:126–36. doi: 10.1016/j.cpr.2017.11.006, PMID: 29195773 PMC6404979

[91] Hui Q Yao C You X. The mechanism of executive dysfunction in depressive symptoms: the role of emotion regulation strategies. Curr Psychol. (2021) 2021. doi: 10.1007/s12144-021-01528-7

[92] Bloch Y Aviram S Faibel N Govezensky J Braw Y Rabany L. The correlation between impaired attention and emotional reactivity in depressed adolescent patients. J Neuropsychiatr Clin Neurosci. (2013) 25:233–6. doi: 10.1176/appi.neuropsych.12080194, PMID: 24026716

[93] Bernerth JB Aguinis H. A critical review and bet-practice recommendations for control variable usage. Pers Psychol. (2016) 69:229–83. doi: 10.1111/peps.12103

[94] Shapiro JR Klein SL Morgan R. Stop ‘controlling’ for sex and gender in global health research. BMJ Glob Health. (2021) 6:e005714. doi: 10.1136/bmjgh-2021-005714, PMID: 33846145 PMC8048018

[95] Donahue JJ McClure KS Moon SM. The relationship between emotion regulation difficulties and psychopathic personality characteristics. Pers Disord Theory Res Treat. (2014) 5:186–94. doi: 10.1037/per0000025, PMID: 24341861

[96] Prado CE Treeby MS Crowe SF. Examining the relationships between sub-clinical psychopathic traits with shame, guilt and externalisation response tendencies to everyday transgressions. J Forens Psychiatry Psychol. (2016) 27:569–85. doi: 10.1080/14789949.2016.1167933

[97] Lanciano T Curci A. Psychopathic traits and self-conscious emotions: what is the role of perspective taking ability? Curr Psychol. (2021) 40:2309–17. doi: 10.1007/s12144-019-0162-2

[98] Lilienfeld SO Latzman RD Watts AL Smith SF Dutton K. Correlates of psychopathic personality traits in everyday life: results from a large community survey. Front Psychol. (2014) 5:740. doi: 10.3389/fpsyg.2014.00740, PMID: 25101019 PMC4106400

[99] Howe J Falkenbach D Massey C. The relationship among psychopathy, emotional intelligence, and professional success in finance. Int J Forensic Ment Health. (2014) 13:337–47. doi: 10.1080/14999013.2014.951103

[100] Brazil KJ Dias CJ Forth AE. Successful and selective exploitation in psychopathy: convincing others and gaining trust. Pers Individ Differ. (2021) 170:110394. doi: 10.1016/j.paid.2020.110394

[101] Hiatt KD Newman JP. Understanding psychopathy: the cognitive side In: Patrick CJ, editor. Handbook of Psychopathy. New York: The Guilford Press (2006). 334–52.

[102] Sellbom M Verona E. Neuropsychological correlates of psychopathic traits in a non-incarcerated sample. J Res Pers. (2007) 41:276–94. doi: 10.1016/j.jrp.2006.04.001

[103] Zeier JD Baskin-Sommers AR Hiatt Racer KD Newman JP. Cognitive control deficits associated with antisocial personality disorder and psychopathy. Pers Disord Theory Res Treat. (2012) 3:283–93. doi: 10.1037/a0023137, PMID: 22452754 PMC3387332

[104] Cotrena C Branco LD Shansis FM Fonseca RP. Executive function impairments in depression and bipolar disorder: association with functional impairment and quality of life. J Affect Disord. (2016) 190:744–53. doi: 10.1016/j.jad.2015.11.007, PMID: 26606718

[105] Dvorak-Bertsch JD Curtin JJ Rubinstein TJ Newman JP. Psychopathic traits moderate the interaction between cognitive and affective processing. Psychophysiology. (2009) 46:913–21. doi: 10.1111/j.1469-8986.2009.00833.x, PMID: 19497014 PMC2746860

[106] Pittenger C Duman RS. Stress, depression, and neuroplasticity: a convergence of mechanisms. Neurpsychopharmacology. (2008) 33:88–109. doi: 10.1038/sj.npp.1301574, PMID: 17851537

[107] Van Praag HM. Can stress cause depression? World J Biol Psychiatry. (2005) 6:5–22. doi: 10.1080/15622970510030018 16166019

[108] Geng J Lei L Han L Gao F. Shyness and depressive symptoms: a multiple mediation model involving core self-evaluations and sense of security. J Affect Disord. (2021) 286:19–26. doi: 10.1016/j.jad.2021.01.035, PMID: 33662715

[109] Watt BD Brooks NS. Self-report psychopathy in an Australian community sample. Psychiatry Psychol Law. (2012) 19:389–401. doi: 10.1080/13218719.2011.585130

