# Supplementary material for: Psychopathic personality traits stress immunity and social potency moderate the relationship between emotional competence and cognitive functions in depression
Source: Front Psychiatry. 2023 Mar 27;14:1061642. doi: 10.3389/fpsyt.2023.1061642 (PMC10084668; doi:10.3389/fpsyt.2023.1061642)
Supplement: Supplementary file 1 [file Data_Sheet_1.docx]

**Supplementary Material**

| **Table 4a.** Correlation analyses between study variables stratified for gender (female participants) | | | | | | | | | | |  |  |  |  |  |  |  |  |
| --- | --- | --- | --- | --- | --- | --- | --- | --- | --- | --- | --- | --- | --- | --- | --- | --- | --- | --- |
| Variable | | 1 | 2 | 3 | 4 | 5 | 6 | 7 | 8 | 9 | 10 | 11 | 12 | 13 | 14 | 15 | 16 | 17 |
| PPI-R | 1. BE | 1 | **.24***** | **-.17*** | **-.29**** | -.16 | **.21**** | **-.15*** | .06 | **-.30***** | -.13 | **-.23***** | **-.14*** | **-.27***** | -.14 | -.04 | -.03 | -.09 |
|  | 2. RN |  | 1 | **.20**** | **.24***** | **.27***** | **.48***** | .11 | **.46***** | -.09 | .08 | -.00 | .11 | .05 | -.04 | .02 | -.04 | -.03 |
|  | 3. SI |  |  | 1 | **.33***** | **-.16*** | -.05 | **.23***** | **.25***** | **.36***** | **.22***** | **.64***** | **.14*** | **.44***** | .04 | .05 | .01 | .04 |
|  | 4. SP |  |  |  | 1 | -.14 | **.26***** | **.19**** | .07 | **.28***** | **.30***** | **.15*** | **.40***** | **.42***** | .11 | .06 | .07 | .10 |
|  | 5. CN |  |  |  |  | 1 | **.16*** | .10 | .12 | **-.29***** | **-.24***** | **-.27***** | .05 | **-.23***** | -.05 | -.14 | -.03 | -.08 |
|  | 6. ME |  |  |  |  |  | 1 | **.21**** | .10 | -.02 | -.07 | -.06 | **.19**** | .04 | -.03 | -.08 | -.03 | -.06 |
|  | 7. CH |  |  |  |  |  |  | 1 | **.14*** | .13 | **-.18**** | .13 | .09 | .06 | -.07 | -.10 | -.04 | -.08 |
|  | 8. F |  |  |  |  |  |  |  | 1 | .00 | -.02 | .04 | -.01 | .00 | .00 | .05 | -.06 | -.01 |
| ECQ | 9. RUE |  |  |  |  |  |  |  |  | 1 | **.43***** | **.44***** | **.51***** | **.82***** | **.15*** | .02 | .08 | .10 |
|  | 10. REO |  |  |  |  |  |  |  |  |  | 1 | **.23***** | **.32***** | **.69***** | .08 | .09 | .01 | .07 |
|  | 11. RCE |  |  |  |  |  |  |  |  |  |  | 1 | .12 | **.57***** | -.04 | .03 | .03 | .01 |
|  | 12. EE |  |  |  |  |  |  |  |  |  |  |  | 1 | **.75***** | **.20**** | -.04 | .09 | .11 |
|  | 13. ECQ Score | |  |  |  |  |  |  |  |  |  |  |  | 1 | **.15*** | .03 | .08 | .11 |
| Cognitive | 14. A/PS |  |  |  |  |  |  |  |  |  |  |  |  |  | 1 | **.37***** | **.73***** | **.89***** |
| Domains | 15. EF |  |  |  |  |  |  |  |  |  |  |  |  |  |  | 1 | **.30***** | **.66***** |
|  | 16. VL/M |  |  |  |  |  |  |  |  |  |  |  |  |  |  |  | 1 | **.86***** |
|  | 17. CogScore | |  |  |  |  |  |  |  |  |  |  |  |  |  |  |  | 1 |
| *Note.* PPI-R = Psychopathic Personality Inventory - Revised. ECQ = Emotional Competence Questionnaire. BE = Blame Externalisation. RN = Rebellious Nonconformity. SI = Stress Immunity. SP = Social Potency. CN = Careless Nonplanfulness. ME = Machiavellian Egocentricity. CH = Coldheartedness. F = Fearlessness. RUE = Recognizing and understanding emotions. REO = Recognizing emotions of others. RCE = Regulation and control of own emotions. EE = Emotional expressivity. A/PS = Attention/Psychomotor speed. EF = Executive function. VL/M = Verbal learning/Memory. *M* = Mean. *SD* = Standard deviation. * *p* < .05. ** *p* < .01. *** *p* < .001. Benjamini-Yekutieli adjustments for all *p*-values. Significant results are printed in bold. Pearson correlation analyses results between study variables indicating non-significant relationships between the Emotional Competence Score (ECQ Score) and the Cognitive Composite Score (CogScore). | | | | | | | | | | | | | | | | | | |

| **Table 4b.** Correlation analyses between study variables stratified for gender male participants) | | | | | | | | | | |  |  |  |  |  |  |  |  |
| --- | --- | --- | --- | --- | --- | --- | --- | --- | --- | --- | --- | --- | --- | --- | --- | --- | --- | --- |
| Variable | | 1 | 2 | 3 | 4 | 5 | 6 | 7 | 8 | 9 | 10 | 11 | 12 | 13 | 14 | 15 | 16 | 17 |
| PPI-R | 1. BE | 1 | **.29***** | **-.29***** | **-.17***** | .12 | **.26***** | -.12 | .10 | **-.34***** | -.07 | **-.30***** | **-.22**** | **-.31***** | -.08 | -.10 | -.01 | -.08 |
|  | 2. RN |  | 1 | **.07** | **.30***** | **.32***** | **.39***** | -.00 | **.34***** | -.04 | .09 | -.07 | .07 | .03 | .01 | .12 | .01 | .05 |
|  | 3. SI |  |  | 1 | **.43***** | **-.18*** | **-.35***** | **.24**** | **.37***** | **.40***** | .01 | **.72***** | **.20*** | **.43***** | .13 | .05 | .05 | .09 |
|  | 4. SP |  |  |  | 1 | -.02 | -.05 | .08 | **.26***** | **.35***** | **.35***** | **.27***** | **.44***** | **.49***** | .09 | .09 | -.05 | .06 |
|  | 5. CN |  |  |  |  | 1 | **.26***** | **.23***** | -.12 | -.15 | -.06 | **-.25**** | .10 | -.11 | -.13 | -.09 | -.17 | -.17 |
|  | 6. ME |  |  |  |  |  | 1 | .14 | -.13 | -.11 | -.05 | **-.35***** | -.12 | **-.21*** | .06 | **.18*** | .07 | .13 |
|  | 7. CH |  |  |  |  |  |  | 1 | .08 | .10 | **-.30***** | .13 | .06 | -.02 | .06 | .04 | .02 | .05 |
|  | 8. F |  |  |  |  |  |  |  | 1 | .18 | .07 | .18 | .09 | .17 | .13 | .09 | .14 | .15 |
| ECQ | 9. RUE |  |  |  |  |  |  |  |  | 1 | **.42***** | **.53***** | **.46***** | **.81***** | **.17*** | .15 | .10 | .17 |
|  | 10. REO |  |  |  |  |  |  |  |  |  | 1 | .09 | **.48***** | **.71***** | .05 | -.01 | -.05 | -.00 |
|  | 11. RCE |  |  |  |  |  |  |  |  |  |  | 1 | **.22*** | **.69***** | .06 | .05 | .03 | .06 |
|  | 12. EE |  |  |  |  |  |  |  |  |  |  |  | 1 | **.77***** | .12 | .04 | -.06 | .04 |
|  | 13. ECQ Score | |  |  |  |  |  |  |  |  |  |  |  | 1 | .14 | .07 | -.00 | .09 |
| Cognitive | 14. A/PS |  |  |  |  |  |  |  |  |  |  |  |  |  | 1 | **.36***** | **.74***** | **.87***** |
| Domains | 15. EF |  |  |  |  |  |  |  |  |  |  |  |  |  |  | 1 | **.38***** | **.70***** |
|  | 16. VL/M |  |  |  |  |  |  |  |  |  |  |  |  |  |  |  | 1 | **.87***** |
|  | 17. CogScore | |  |  |  |  |  |  |  |  |  |  |  |  |  |  |  | 1 |
| *Note.* PPI-R = Psychopathic Personality Inventory - Revised. ECQ = Emotional Competence Questionnaire. BE = Blame Externalisation. RN = Rebellious Nonconformity. SI = Stress Immunity. SP = Social Potency. CN = Careless Nonplanfulness. ME = Machiavellian Egocentricity. CH = Coldheartedness. F = Fearlessness. RUE = Recognizing and understanding emotions. REO = Recognizing emotions of others. RCE = Regulation and control of own emotions. EE = Emotional expressivity. A/PS = Attention/Psychomotor speed. EF = Executive function. VL/M = Verbal learning/Memory. *M* = Mean. *SD* = Standard deviation. * *p* < .05. ** *p* < .01. *** *p* < .001. Benjamini-Yekutieli adjustments for all *p*-values. Significant results are printed in bold. Pearson correlation analyses results between study variables indicating non.significant relationships between the Emotional Competence Score (ECQ Score) and the Cognitive Composite Score (CogScore). | | | | | | | | | | | | | | | | | | |

**Moderation analyses assumption calculations**

**1. Linearity, Homoscedasticity and Normality of Residuals**

All three assumptions were checked by a graphical examinations of (a) the “residuals vs fitted”- and “scale-location”-plot, indicating linearity and homoscedasticity and (b) the “normal q-q”-plot, indicating normality of residuals.

**2. Independence of residuals**

Independence of residuals was tested using the Durbin-Watson test. Values should be around 2 for indepencence of residuals.

**3. Outliers with leverage**

To determine outliers with leverage, we used the Cook’s distance. It is indicated that data exceeding the Cook’s distance threshold of > 1 signify an outlier with leverage (Field, 2018). Our analyses indicated no such outliers (all Cook’s distance values below 1).

**4. Multicollinearity**

To check for multicollinearity, we used the Variance-Inflation-factor. This value should not exceed 10.

**Model a) ECQ, Cognition Score and the PPT Blame Externalisation**

**1. Linearity, Homoscedasticity and Normality of Residuals**


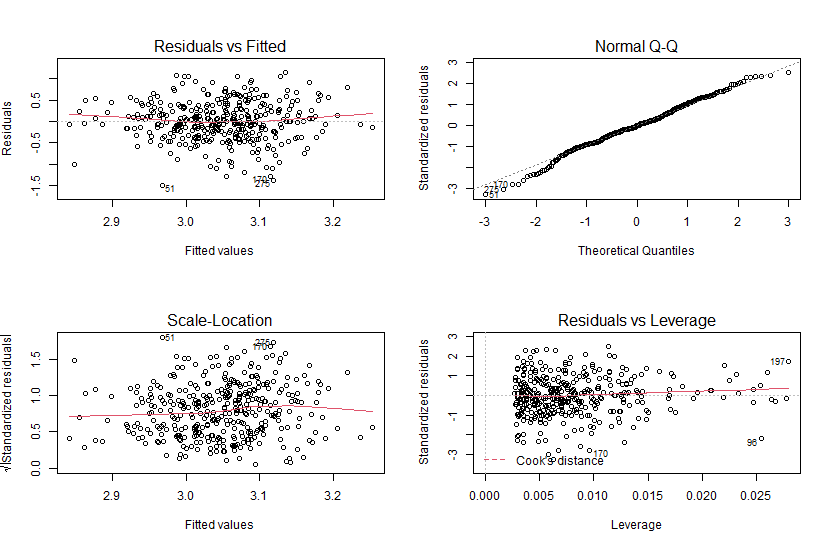


**2. Independence of residuals**

Durbin-Watson-Coefficient: 1.92

**3. Outliers with leverage**

Three cases exceeding three SD above mean found.

| Case 51 | 0.04330774 |
| --- | --- |
| Case 275 | 0.04323970 |

Graphical illustration of the outliers can be found in the boxplot.


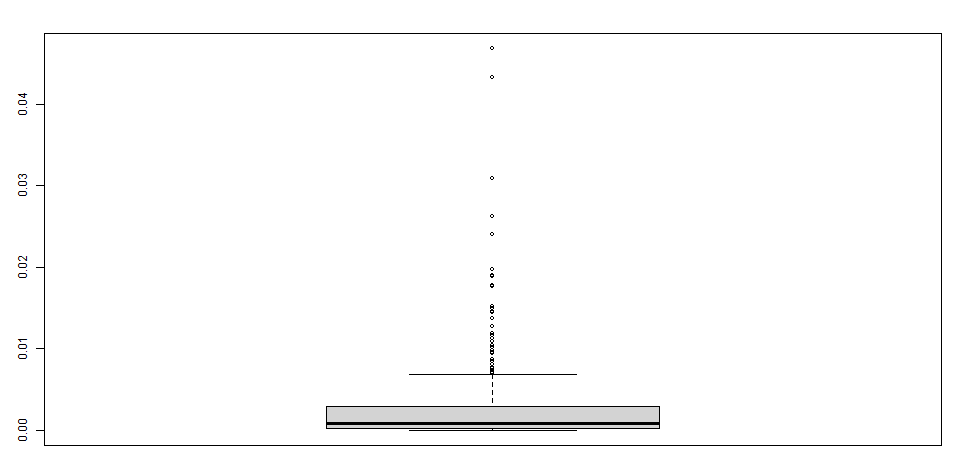


**4. Multicollinearity**

VIF = 1.01 (for both the Cognition Score and the PPT Blame Externalisation)

**Model b) ECQ, Cognition Score and the PPT Rebellious Nonconformity**

**1. Linearity, Homoscedasticity and Normality of Residuals**


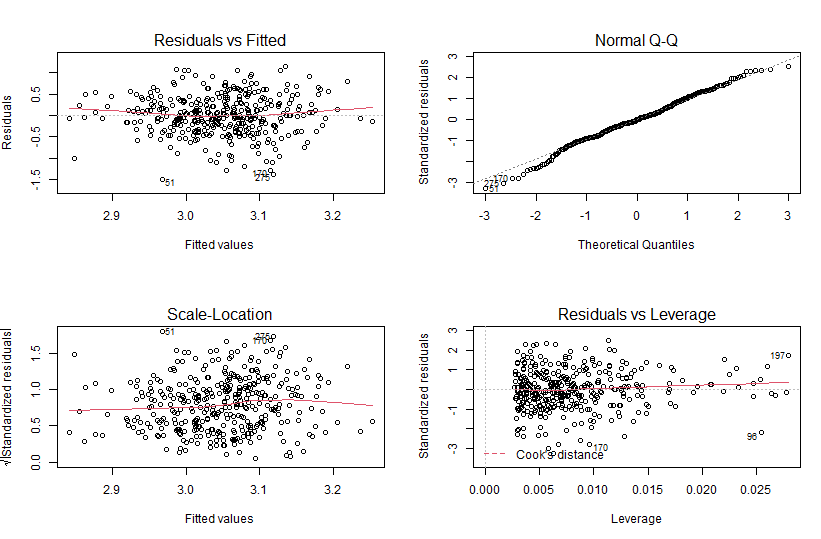


**2. Independence of residuals**

Durbin-Watson-Coefficient: 1.93 (for both the Cognition Score and the PPT Rebellious Nonconformity)

**3. Outliers with leverage**

Two cases exceeding three SD above mean found.

| Case 51 | 0.05394708 |
| --- | --- |
| Case 275 | 0.01980430 |

Graphical illustration of the outliers can be found in the boxplot.


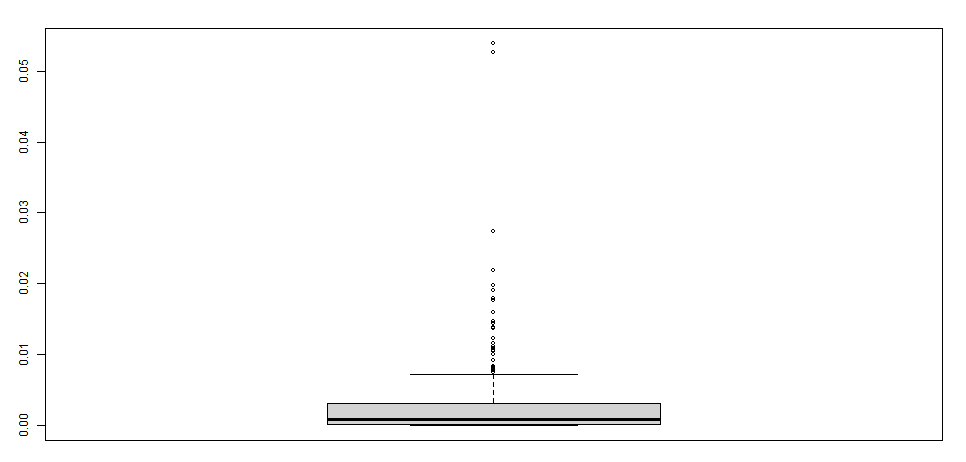


**4. Multicollinearity**

VIF = 1.00 (for both the Cognition Score and the PPT Rebellious Nonconformity)

**Model c) ECQ, Cognition Score and the PPT Stress Immunity**

**1. Linearity, Homoscedasticity and Normality of Residuals**
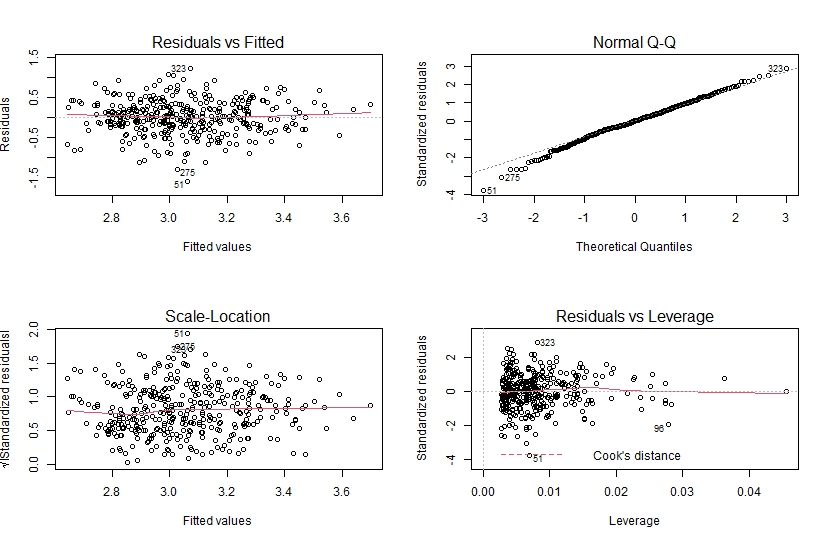


**2. Independence of residuals**

Durbin-Watson-Coefficient: 1.94

**3. Outliers with leverage**

Two cases exceeding three SD above mean found.

| Case 51 | 0.03311564 |
| --- | --- |
| Case 275 | 0.02004890 |

Graphical illustration of the outliers can be found in the boxplot.


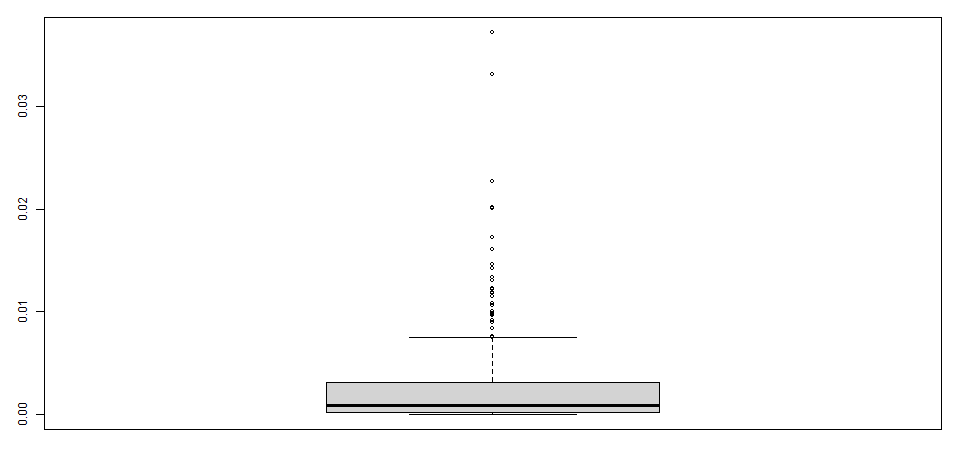


**4. Multicollinearity**

VIF = 1.00 (for both the Cognition Score and the PPT Stress Immunity)

**Model c) ECQ, Cognition Score and the PPT Social Potency**

**1. Linearity, Homoscedasticity and Normality of Residuals**


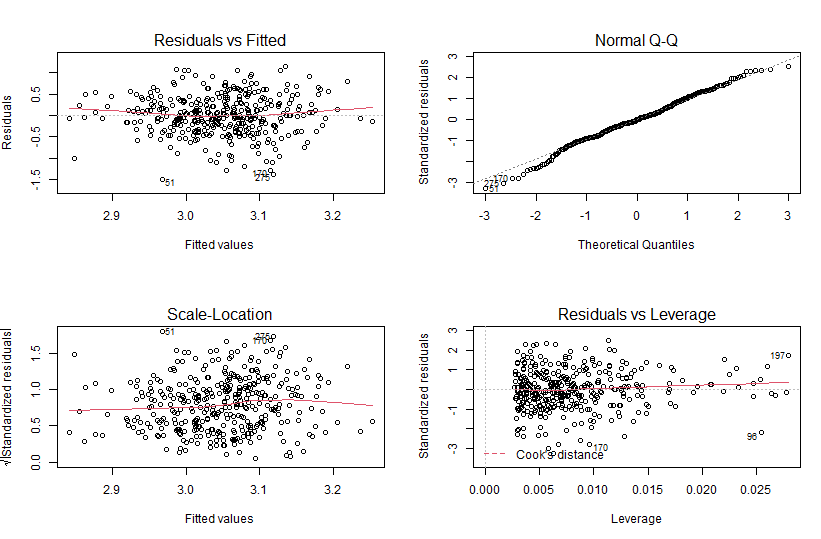


**2. Independence of residuals**

Durbin-Watson-Coefficient: 1.93

**3. Outliers with leverage**

Two cases exceeding three SD above mean found.

| Case 51 | 0.02764528 |
| --- | --- |
| Case 170 | 0.01465374 |

Graphical illustration of the outliers can be found in the boxplot.


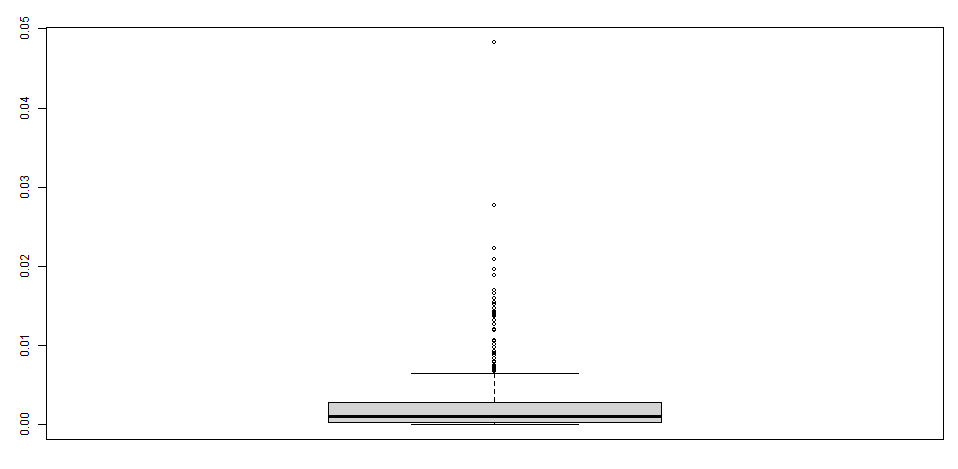


**4. Multicollinearity**

VIF = 1.00 (for both the Cognition Score and the PPT Social Potency)

**Model d) ECQ, Cognition Score and the PPT Coldheartedness**

**1. Linearity, Homoscedasticity and Normality of Residuals**


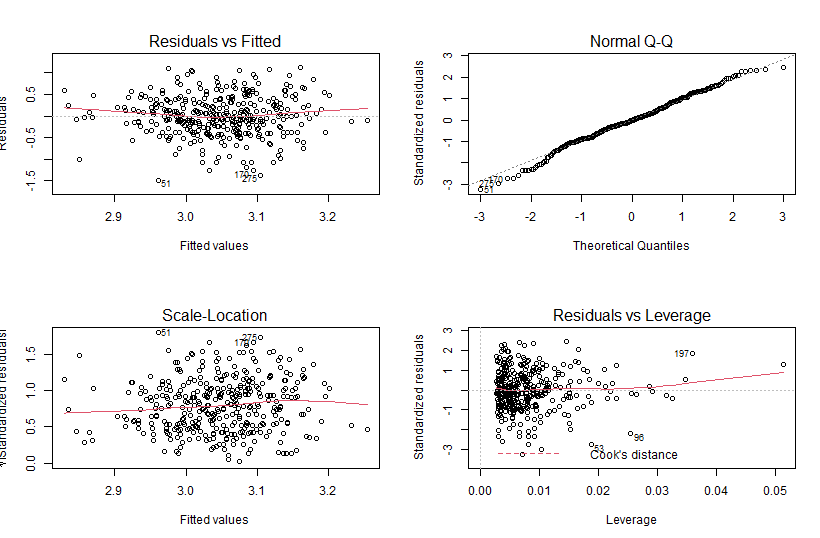


**2. Independence of residuals**

Durbin-Watson-Coefficient: 1.93

**3. Outliers with leverage**

One case exceeding three SD above mean found. However, Cook’s distance values did not exceed 1.

| Case 51 | 0.02524407 |
| --- | --- |

Graphical illustration of the outlier can be found in the boxplot.


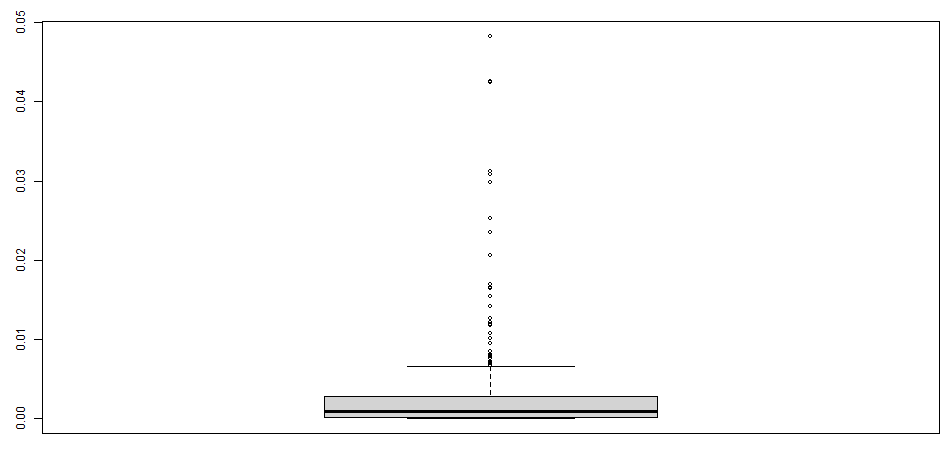


**4. Multicollinearity**

VIF = 1.00 (for both the Cognition Score and the PPT Coldheartedness)

**Model e) ECQ, Cognition Score and the PPT Machiavellianism Egocentricity**

**1. Linearity, Homoscedasticity and Normality of Residuals**


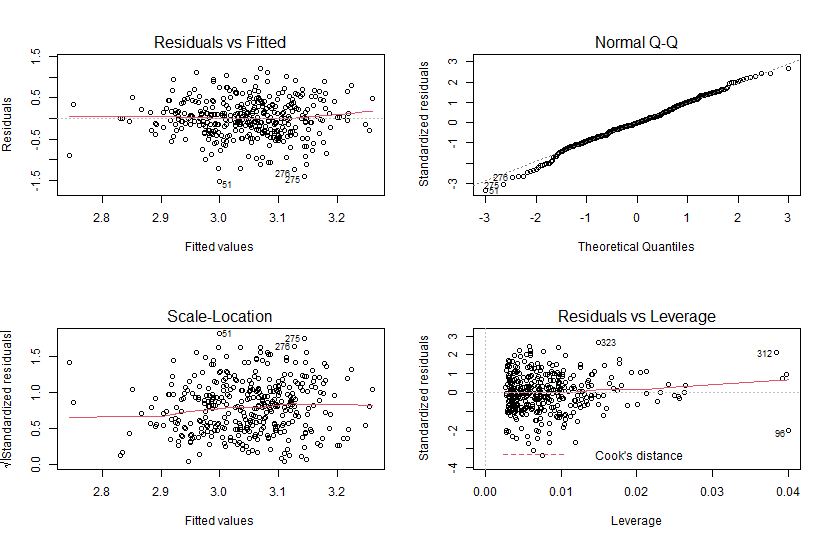


**2. Independence of residuals**

Durbin-Watson-Coefficient: 1.94

**3. Outliers with leverage**

Two cases exceeding three SD above mean found.

| Case 51 | 0.02821825 |
| --- | --- |
| Case 275 | 0.0024583 |

Graphical illustration of the outliers can be found in the boxplot.


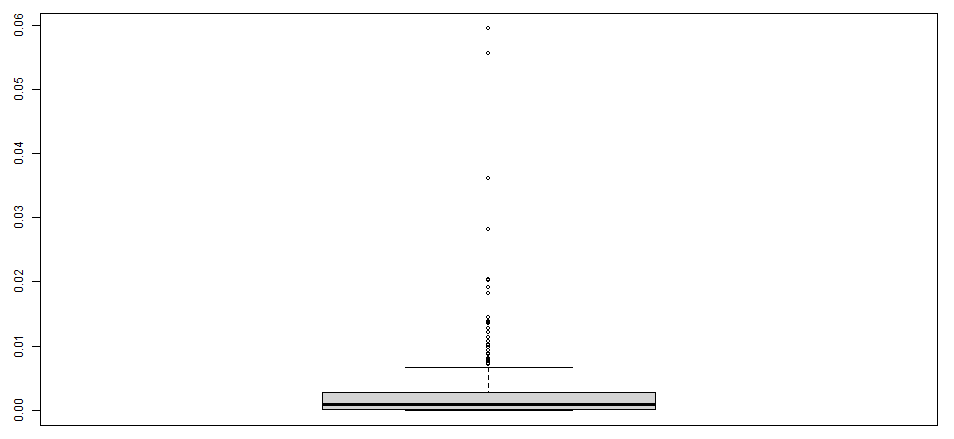


**4. Multicollinearity**

VIF = 1.00 (for both the Cognition Score and the PPT Machiavellianism Egocentricity)

**Model f) ECQ, Cognition Score and the PPT Careless Nonplanfulness**

**1. Linearity, Homoscedasticity and Normality of Residuals**


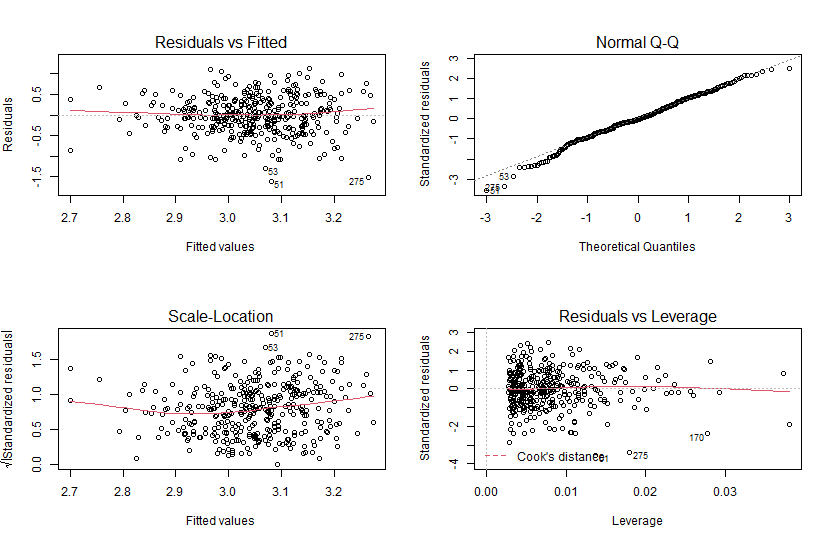


**2. Independence of residuals**

Durbin-Watson-Coefficient: 1.90

**3. Outliers with leverage**

Two cases exceeding three SD above mean found.

| Case 51 | 0.05830549 |
| --- | --- |
| Case 275 | 0.06957943 |

Graphical illustration of the outliers can be found in the boxplot.


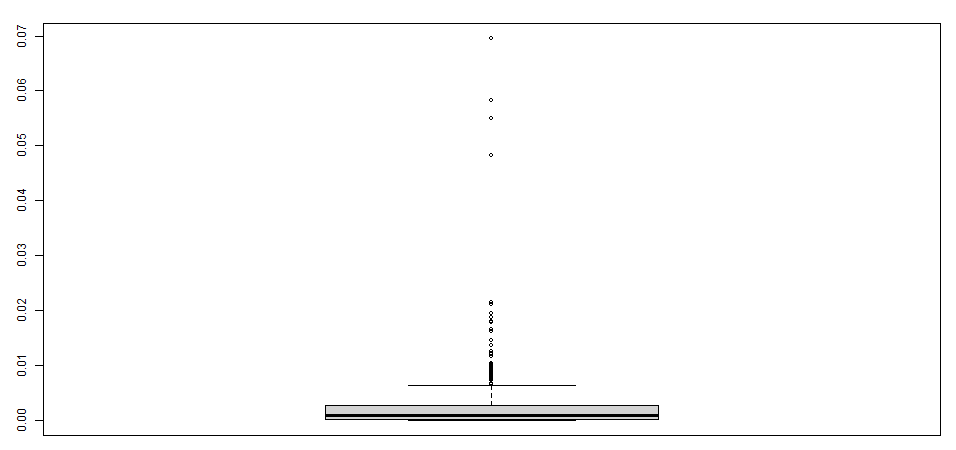


**4. Multicollinearity**

VIF = 1.01 (for both the Cognition Score and the PPT Careless Nonplanfulness)

**Model g) ECQ, Cognition Score and the PPT Fearlesness**

**1. Linearity, Homoscedasticity and Normality of Residuals**


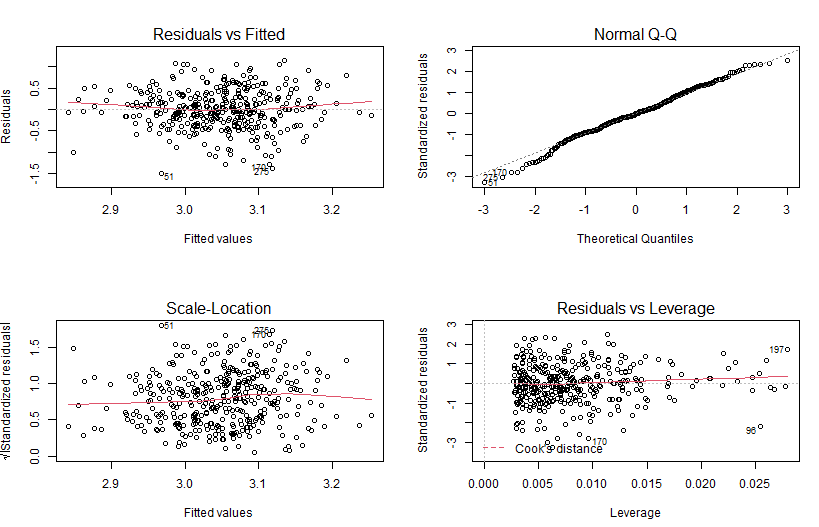


**2. Independence of residuals**

Durbin-Watson-Coefficient: 1.93

**3. Outliers with leverage**

Two cases exceeding three SD above mean found.

| Case 51 | 0.02214214 |
| --- | --- |
| Case 275 | 0.01754514 |

Graphical illustration of the outliers can be found in the boxplot.


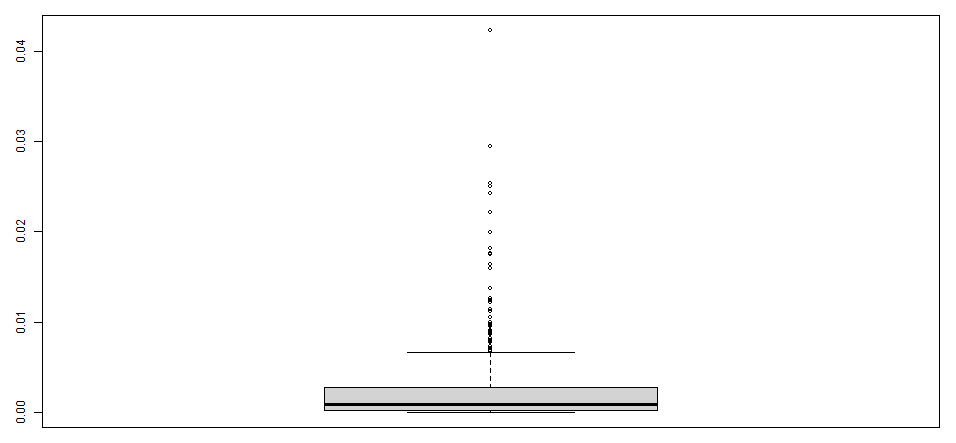


**4. Multicollinearity**

VIF = 1.00 (for both the Cognition Score and the PPT Careless Nonplanfulness)

**Moderation analyses excluding outliers deviating more than three standard deviations**

**Exclusion of cases 51, 170, 275**

| **Table 5a.** Moderation analyses results for psychopathic personality traits scores moderating the relationship between emotional competence (ECQ Score) and cognitive composite score | | | | | | | | |
| --- | --- | --- | --- | --- | --- | --- | --- | --- |
| Effect | | Estimate | *SE* | *t* | | 95% CI | | *p* |
|  | |  |  |  | | *LL* | *UL* |  |
|  | Blame Externalisation (BE) | | | | | | |  |
| Constant | | 0.06 | .04 | 1.35 | | -0.03 | 0.14 | .177 |
| ECQ Score | | **0.22*** | .10 | 2.29 | | 0.03 | 0.41 | .022 |
| BE | | -0.01 | .01 | -1.25 | | -0.02 | 0.00 | .211 |
| ECQ Score x BE | | -0.01 | .01 | -1.25 | | -0.03 | 0.01 | .213 |
|  | |  | | | Rebellious Nonconformity (RN) | | |  |
| Constant | | 0.08 | .04 | 1.77 | | -0.01 | 0.16 | .078 |
| ECQ Score | | **0.25**** | .09 | 2.74 | | 0.08 | 0.43 | .001 |
| RN | | -0.00 | .00 | -0.64 | | -0.01 | 0.01 | .523 |
| ECQ Score x RN | | 0.01 | .01 | 0.63 | | -0.01 | 0.02 | .534 |
|  | Stress Immunity (SI) | | | | | |  |  |
| Constant | | 0.04 | .04 | 0.87 | | -0.05 | 0.13 | .384 |
| ECQ Score | | **0.29**** | .10 | 2.88 | | 0.09 | 0.49 | .004 |
| SI | | -0.01 | .01 | -1.05 | | -0.02 | 0.01 | .293 |
| ECQ Score x SI | | **0.03*** | .01 | 2.16 | | 0.00 | 0.05 | .031 |
|  | Social Potency (SP) | | | | | | |  |
| Constant | | 0.04 | .05 | 0.90 | | -0.05 | 0.13 | .366 |
| ECQ Score | | **0.25 *** | .10 | 2.40 | | 0.05 | 0.44 | .017 |
| SP | | 0.00 | .01 | 0.05 | | -0.01 | 0.01 | .972 |
| ECQ Score x SP | | **0.02*** | .01 | 1.97 | | 0.00 | 0.03 | .049 |
|  | |  | | | Coldheartedness (CH) | | |  |
| Constant | | 0.07 | .04 | 1.75 | | -0.01 | 0.16 | .080 |
| ECQ Score | | **0.26**** | .09 | 279 | | 0.07 | 0.44 | .001 |
| CH | | -0.01 | .01 | -1.69 | | -0.03 | 0.00 | .092 |
| ECQ Score x CH | | -0.01 | .01 | -0.59 | | -0.04 | 0.02 | .553 |
|  | Machiavellian Egocentricity (ME) | | | | | | |  |
| Constant | | 0.08 | .04 | 1.89 | | -0.00 | 0.16 | .059 |
| ECQ Score | | **0.25**** | .09 | 2.71 | | 0.01 | 0.43 | .006 |
| ME | | 0.00 | .01 | 0.24 | | -0.01 | 0.02 | .810 |
| ECQ Score x ME | | 0.02 | .02 | 1.36 | | -0.01 | 0.05 | .174 |
|  | Careless Nonplanfulness (CN) | | | | | | |  |
| Constant | | 0.08 | .04 | 1.76 | | -0.01 | 0.16 | .079 |
| ECQ Score | | **0.23**** | .09 | 2.44 | | 0.05 | 0.42 | .015 |
| CN | | -0.01 | .01 | -1.77 | | -0.03 | 0.00 | .077 |
| ECQ Score x CN | | 0.00 | .01 | -0.05 | | -0.04 | 0.04 | .956 |
|  | Fearlessness (F) | | | | | | |  |
| Constant | | 0.07 | .04 | 1.76 | | -0.01 | 0.16 | .078 |
| ECQ Score | | **0.26**** | .09 | 2.80 | | 0.08 | 0.44 | .005 |
| F | | -0.01 | .01 | -0.75 | | -0.02 | 0.01 | .454 |
| ECQ Score x F | | -0.00 | .02 | -0.16 | | -0.04 | 0.03 | .868 |
| *Note*. Estimate = Unstandardized regression weight. *SE* = Standard error of the unstandardized regression weight. 95% CI = 95% - Confidence intervals based on 10000 samples (BCa bootstrapping). *LL* = Lower level of the 95% confidence interval. *UL* = Upper level of the 95% confidence interval. * *p* < .05, ** *p* <.01. Significant results are printed in bold. Results indicate a significant interaction effect between the ECQ score and the cognitive composite score, which is positively moderated by SI and SP. | | | | | | | | |
|  | | | | | | | | |
