# Supplementary material for: Psychopathic personality traits stress immunity and social potency moderate the relationship between emotional competence and cognitive functions in depression
Source: Front Psychiatry. 2023 Mar 27;14:1061642. doi: 10.3389/fpsyt.2023.1061642 (PMC10084668; doi:10.3389/fpsyt.2023.1061642)
Supplement: Supplementary file 2 [file Presentation_1.pdf]

Do psychopathic personality traits affect the relationship between cognition and emotion in depression?

Sample

Rehabilitation center for affective and stress-related disorders, Austria

373

Individuals with depression

n = 214

n = 158

Methods

Self-assessment questionnaires

for

- Emotional competences
- Psychopathic personality traits

Neuropsychological testbattery

for

Cognition
- Attention/processing speed
- Verbal memory/language
- Executive function

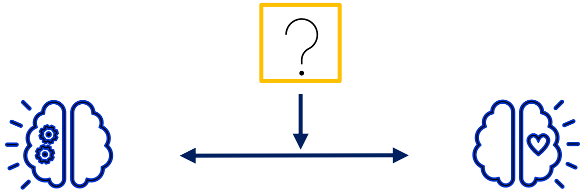

Results

Relationship between cognition and emotion is positively moderated by:

- Psychopathic Stress Immunity
- Psychopathic Social Potency

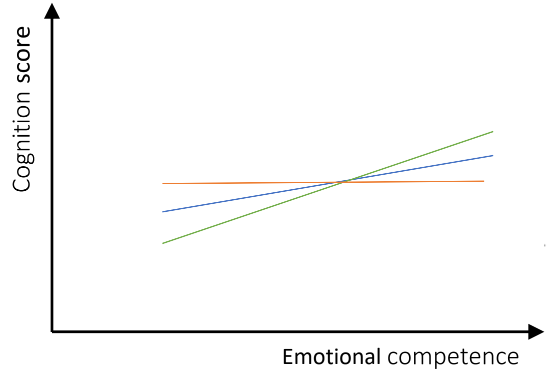

Conclusion

- Some psychopathic traits have a positive effect on deteriorated emotion and cognition in depression
- Personality traits should be identified as resilience-strengthening or deteriorating factors in depression

Graphical abstract by Schönthaler et al., 2023
